# Chronic corticosterone-mediated dysregulation of microRNA network in prefrontal cortex of rats: relevance to depression pathophysiology

**DOI:** 10.1038/tp.2015.175

**Published:** 2015-11-17

**Authors:** Y Dwivedi, B Roy, G Lugli, H Rizavi, H Zhang, N R Smalheiser

**Affiliations:** 1 Department of Psychiatry and Behavioral Neurobiology, UAB Mood Disorder Program, University of Alabama at Birmingham, Birmingham, AL, USA; 2 Department of Psychiatry, University of Illinois at Chicago, Chicago, IL, USA

## Abstract

Stress plays a major role in inducing depression, which may arise from interplay between complex cascades of molecular and cellular events that influence gene expression leading to altered connectivity and neural plasticity. In recent years, microRNAs (miRNAs) have carved their own niche owing to their innate ability to induce disease phenotype by regulating expression of a large number of genes in a cohesive and coordinated manner. In this study, we examined whether miRNAs and associated gene networks have a role in chronic corticosterone (CORT; 50 mg  kg^−1^ × 21 days)-mediated depression in rats. Rats given chronic CORT showed key behavioral features that resembled depression phenotype. Expression analysis revealed differential regulation of 26 miRNAs (19 upregulated, 7 downregulated) in prefrontal cortex of CORT-treated rats. Interaction between altered miRNAs and target genes showed dense interconnected molecular network, in which multiple genes were predicated to be targeted by the same miRNA. A majority of altered miRNAs showed binding sites for glucocorticoid receptor element, suggesting that there may be a common regulatory mechanism of miRNA regulation by CORT. Functional clustering of predicated target genes yielded disorders such as developmental, inflammatory and psychological that could be relevant to depression. Prediction analysis of the two most prominently affected miRNAs miR-124 and miR-218 resulted into target genes that have been shown to be associated with depression and stress-related disorders. Altogether, our study suggests miRNA-mediated novel mechanism by which chronic CORT may be involved in depression pathophysiology.

## Introduction

Depression is one of the most prevalent psychiatric disorders worldwide and is a major public health concern.^1^ About 50% patients suffering from depression show suicidal thought and tendency at some point in their lives; of them 10–15% eventually commit suicide.^2^ Interestingly, almost 50% of patients do not recover following an antidepressant trial and 20% of these patients fail to respond to any intervention.^3, 4^ This could partially be a result of a poor understanding of the molecular pathophysiology underlying depression.

Several lines of evidence suggest that depression arises from a combination of genetic and environmental factors.^5^ Stress represents one of the major environmental risk factors that can lead to precipitation of depression.^6^ How stress causes depressive symptomatology is not well-defined, nevertheless, there is a well-established connection of stress-mediated hyperactive hypothalamus–pituitary–adrenal axis (HPA) and depression, which is primarily associated with altered expression and function of glucocorticoid receptors (GRs) that may lead to feed-back inhibition, resulting in elevated levels of circulating glucocorticoids and protracted responses to stressors.^6^ Recently, small non-coding RNAs, which act as mega controllers of gene expression, have gained a wide-spread attention as a major regulator of disease phenotypes.^7, 8^ These small non-coding RNAs regulate gene expression by several mechanisms including ribosomal RNA modifications, repression of mRNA expression by RNA interference, alternative splicing and regulatory mechanisms mediated by RNA–RNA interactions.^9^ The group of small non-coding RNAs includes: microRNAs (miRNAs), small nucleolar RNAs, small interfering RNAs, piwi-interacting RNAs, splisosomal RNAs and endoribonucleases RNase mitochondrial RNA processing (MRP) and RNase P genes. Of them, miRNAs are the most studied and well-characterized non-coding RNAs and have emerged as a most prominent regulator of neural plasticity and higher brain functioning.^8, 10, 11^

miRNAs are encoded in the genomes (inter or intragenic) and are transcribed into primary miRNA gene transcripts that are converted into precursor miRNAs by Drosha, a nuclear RNase III. Precursor miRNAs are then exported to cytosol and processed by the RNase III Dicer to generate mature miRNAs, which are about ~22 nt in length. These mature miRNAs are then incorporated into the RNA-induced silencing complex, which then regulate gene expression by pairing primarily to the 3′ untranslated region of protein-coding mRNAs to repress target mRNA translation and/or induce target degradation.^12^ Generally, the gene expression regulation by miRNAs occurs in a coordinated and cohesive manner. Because of this feature, miRNAs are able to regulate entire genetic circuitries and thereby play a critical role in maintaining biological homeostasis.^13^ Considering the fundamental role of miRNAs in mediating biological events, any perturbations in the expression of miRNAs may result in the imbalance of homeostasis, which are often reflection of imbalances in the regulatory network that can distinguish normal vs disease states. Hence, miRNA fingerprinting is currently being utilized as novel tool for diagnosis, prognosis and disease surveillance.

In recent years, miRNAs have been under intense investigation for their role in psychiatric disorders including major depression. These include studies in human postmortem brain and fibroblasts of depressed patients as well as in brains of animals that show resiliency to chronic stress.^14, 15, 16^ In this context, we recently reported that miRNAs are highly dysregulated in the dorsolateral prefrontal cortex of depressed individuals^17^ and hopeless behavior in rats can cause blunted miRNA response in frontal cortex.^18^ Also, we found that depression phenotype in rats was reversed by a fluoroquinoline compound that acts on dicer/TRBP complex.^19^ To further understand the role of miRNAs in stress and depression pathophysiology, we examined miRNA expression and mapped network of genes based on *in silico* prediction model regulated by miRNAs in prefrontal cortex (PFC) of rats given chronic administration of exogenous corticosterone (CORT) as a means to study the elevated CORT levels that would occur as a consequence to stress exposure and that can lead to depressive phenotype.^20^

## Materials and methods

### Animals

Virus-free male Sprague-Dawley rats (Harlan Sprague-Dawley Laboratories, Indianapolis, IN, USA) were housed under standard laboratory conditions (temperature 21±1 °C, humidity 55±5%, 12:12-h light/dark cycle). Animals were provided free access to food and water and adapted to the laboratory environment for 1 week before the experiment. Body weights were 325–350 g at the start of the experiment. Rats were housed in groups of three. Experimental procedures were approved by the Animal Care Committee of the University of Illinois at Chicago.

### CORT injections

A total of 64 animals were randomly assigned to 2 groups: 1 group was administered CORT (Sigma Chemical, St. Louis, MO, USA) s.c. once per day in a dose of 50 mg  kg^−1^ of body weight, emulsified in propylene glycol (Fisher Scientific, Pittsburgh, PA, USA). The other group of animals was injected with vehicle (propylene glycol). Injections were given between 0900 and 1100 hours for 21 consecutive days. This dose was selected based on previous studies in which similar dose was effective in inducing depression-like behavior in rats.^21, 22, 23, 24^ Separate groups of rats underwent behavioral testing independently to avoid any confounding effects (*n*=8 per group). We chose *n*=8 per group based on previous studies in which similar CORT dose produced robust depressive behavior phenotype using 8–15 animals per group.^19, 20^ In certain cases, CORT was given for 21 consecutive days prior to the behavioral testing and were continued through behavioral testing. miRNA expression was examined in pooled brain samples from a separate set of rats 24 h after the last CORT injection to avoid any stress-associated confounding changes. Rats were decapitated 24 h after the last CORT injection between 0900  and 1100 hours, corresponding to 3–5 h after lights on. The trunk blood was collected on ice and was centrifuged, and then the serum was stored at −80 °C until the assays were performed. Serum CORT levels were measured by a radioimmunoassay kit (ICN Biomedical, Irvine, CA, USA). For miRNA assays, brains were removed quickly and were dissected on ice as follows: first, the cerebellum was removed; then the anterior parts of the frontal lobes were dissected on the level of bregma 3.2 in accordance with Paxinos and Watson coordinates^25^ after removal of their basal parts at the level of the rhinal fissures. The collected parts were regarded as PFC and immediately frozen on dry ice before transferring to −80 °C for storage. Body weight was recorded on day 1, 7, 14 and 21. Adrenal weight was determined on day 21.

### Behavioral procedures

The experimenters were blinded to all the behavioral measurements. The number of animals used in each experiment is provided in Supplementary Table 1.

### Sucrose preference test

Sucrose preference test was performed as described by Mao *et al.*^26^ Initially, the rats were trained to adapt to sucrose solution (1%, w/v) by placing two bottles of sucrose solution in each cage for 24 h; then one of the bottles was replaced with water for 24 h. After the adaptation procedure, the rats were deprived of water and food for 24 h. The rats were housed in individual cages and given free access to the two bottles containing 100 ml of sucrose solution (1%, w/v) and 100 ml of water, respectively. After 3 h, the volumes of consumed sucrose solution and water were recorded. The percentage of sucrose solution from the total liquid ingested represented the parameter of hedonic behavior.

### Forced swim test

The forced swim test (FST) was performed essentially by the procedure described by O'Donovan *et al.*^27^ The procedure was done in 2 days. The FST involved a 15-min pre-test followed by a 5 min test 24 h later. The test was conducted in a rectangular Plexiglas swim tank (25-cm long × 25-cm wide × 60-cm high). The tank was filled with 27±2 °C water to a depth of 30 cm and rats were placed individually for 5 min. The length of time animals spent immobile was measured over the 5-min test session. Immobility was defined as moving the limbs only enough to stay above water, as opposed to escaping or exploring behavior.

### Open-field test

The open-field test was used to assess general locomotor activity as described by Marks *et al.*^24^ The open field was a 70-cm long × 70-cm wide × 60-cm high black wooden box with a transparent Plexiglas bottom and no top. The floor of the open field was divided into 36 identical squares by tape attached underneath the floor. Each rat was placed individually into a corner of the open field and allowed to explore for 5 min. Number of lines crossed during the 5 min session was calculated. Locomotor activity was inferred from the number of lines crossed.

### RNA isolation

Total RNA was isolated from PFC using a modified protocol designed to optimize recovery of small RNAs.^28^ Glycoblue 20 μg (Ambion, Waltham, MA, USA) was added to the RNA precipitation step, which was allowed to proceed overnight at −20 °C. The RNA pellet was spun down at 20 000 *g* for 25 min at 4 ^o^C; rinsed with 80% ethanol in nuclease-free water (Invitrogen Life Technologies, Carlsbad, CA, USA); resuspended in RNAsecure (Ambion); and treated with DNase I using DNA-free kit (Ambion). The purity of RNA was determined by measuring the optical density with an absorbance ratio of 260/280 (NanoDrop spectrophotometer, ThermoScientific, Waltham, MA, USA) and running the samples on agarose gel to determine the integrity.

### TLDA-based miRNA expression analysis

Expression of miRNAs was determined as described earlier.^18^ Reverse transcription (RT) was performed following the manufacturer's protocol with the TaqMan MiRNA Reverse Transcription kit (Applied Biosystems, Foster City, CA, USA) and the multiplex RT for TaqMan MicroRNA Assays that consisted of eight predefined RT primer pools. For each RT pool, 100 ng of total RNA was used and the product was diluted 1:62.5 and 55 μl diluted product mixed with 55 μl of TaqMan Universal PCR Master Mix (Applied Biosystems), No AmpErase UNG (Applied Biosystems). About 100 μl of each mix was dispensed in the appropriate well in the TaqMan Rodent MicroRNA Array v3.0 (Taqman low density array (TLDA), Applied Biosystems) and run to 40 cycles as per manufacturer's protocol on an ABI 7900HT RT–PCR machine (Applied Biosystems). miRNAs were assayed on two plates, A and B; A plates contained many of the canonical miRNA sequences in miRBase, whereas the B plate primarily contained minor or star (*) miRNAs sequences arising from the opposite arm of the pre-miR hairpin precursor (Supplementary Table 2). A sample processed without RT showed no detectable miRNA values. Using samples run on duplicate plates to monitor inter-plate reliability, we observed that Ct values >35 were less reliable, and so Ct=35 was set as the threshold of detectability (Supplementary Table 2). Median values (miRNAs and small RNAs) of each replicate was determined and used for normalization. We also checked geometric means of endogenous genes provided within the TLDA plates (U6, Y1 and U87). The geometric means of these endogenous RNAs, which is represented as Ct values, did not change between vehicle controls and CORT-treated groups (Vehicle: 21.82±0.28; CORT treated: 21.58±0.27). Plant-specific ath-miR-159a was also included in the TLDA plate as negative control, which did not show any expression in rat PFC. Fold-differences in miRNA expression across groups was calculated following ΔΔCt method (shown in Supplementary Table 3).

### Statistical analysis

All data were analyzed using Statistical Package for the Social Sciences, version 21 (IBM, New York, NY, USA) and are represented as means±s.d. None of the animals were excluded from the statistical analysis. Group differences in body weight were analyzed using analysis of variance-repeated measures to test for the effects of time and CORT treatment, as well as their interactions. This was followed by *post hoc t*-tests. Serum CORT levels and behavioral tests between vehicle and CORT-treated groups were analyzed by independent sample *t*-test. Statistical significance was calculated using both the non-parametric Wilcoxon paired sign-rank test, two-tailed and the paired *t*-test. Paired tests were conducted to match rats that were subjected to CORT and vehicle treatment in the same experiments and samples that were prepared and analyzed for miRNA abundance in the same runs.^28^ Both methods produced similar results. The criterion for statistical significance was set at *P*⩽0.05.

### Target genes and network analysis

Statistically significant miRNAs were analyzed for their mRNA targets using Ingenuity Pathway Analysis Software (IPA; Qiagen, Valencia, CA, USA). Briefly the miRNA-target module in IPA was used to filter the list of significantly altered up or downregulated miRNAs to retrieve a population of target mRNAs. Two sets of target mRNAs were analyzed: (1) a set of mRNA target gene was prepared using TarBase, Ingenuity Expert Finding and miRecords (Qiagen, Redwood City, CA, USA) with experimental validation; (2) another set of mRNA target gene was prepared based on number of conserved targeting site and total context score prediction value with a high-to-moderate degree of 3′ untranslated region-binding specificity with the miRNA seed sequence using TargetScan (Cambridge, MA, USA). The short listed target genes were further analyzed with IPA core analysis module for functional enrichment of target genes deciphering their role in canonical pathway, molecular network along with disease pathway using Fisher Exact Test and *P*-value threshold set at ⩽0.05. The initial data output from canonical pathway were further filtered by setting the criteria stringently to represent only a few selected pathways related to stress pathophysiology.

### Validation analysis of select miRNAs by qPCR

Relative quantification of select mature miRNAs was reexamined following a poly-A tailing method using Poly-A polymerase kit (Life Technologies, Carlsbad, CA, USA). Polyadenylation of short RNAs was carried out by 4 U of *Escherichia coli* poly-A polymerase enzyme in addition to 1x poly-A polymerase buffer, 2.5 mM MnCl_2_, 1 mM rATP and 40 U of RNaseOut (Life Technologies) for a 10 μl reaction volume. The reaction mixture was initially incubated for 30 min at 37 °C followed by the addition of 1 μm oligo dT adapter primer (5′**-**GCGAGCACAGAATTAATACGACTCACTATAGGTTTTTTTTTTTTTTTTTTVN-3′) and then quick incubation of 5 min at 60 °C. Reaction was finally cooled to 4 °C before proceeding to complementary DNA synthesis. The poly-A tailed RNA population was reverse transcribed following M-MLV RT-mediated 1st strand complementary DNA synthesis method mentioned in manufacturer's protocol (Life Technologies).

Relative transcript abundance of specific miRNA was quantified using EvaGreen in the MX3005P qPCR system (Stratagene, Santa Clara, CA, USA). Briefly, mature miRNAs were amplified using 1 × EvaGreen qPCR Mastermix (Applied Biological Material, Richmond, BC, Canada) in combination with 0.8 μM each of gene-specific forward primer and oligo dT adapter sequence specific universal reverse primer. The primer sequences were as follows: miR-124: 5′-TAAGGCACGCGGTGAAT-3′ miR-218: 5′-TTGTGCTTGATCTAACCATGTAAAA-3′ miR-29a: 5′-TAGCACCATCTGAAATCGGTTA-3′ miR-146a: 5′-TGAGAACTGAATTCCATGGGTT-3′ miR-200c: 5′-CTGCCGGGTAATGATGGA-3′ miR-155: 5′-TTAATGCTAATTGTGATAGGGGTAAAA-3′. U6 small nuclear RNA-specific forward (5′-CTCGCTTCGGCAGCACA-3′) and reverse (5′-AACGCTTCACGAATTTGCGT-3′) primers were used at 0.8 μM concentration to amplify endogenous control transcript. Fortyfold diluted raw complementary DNA was used as a template for quantitative real-time PCR (qPCR) amplification following a thermal parameter of initial denaturation at 95 °C for 10 min followed by repeating 40 cycles of denaturation at 95 °C for 10 s, primer annealing at 55 °C for 15 s and an extension of amplicon at 72 °C for 20 s. Possibility of primer dimer formation and secondary product amplification was ruled out by running a single cycle of EvaGreen-specific dissociation curve analysis program with initial denaturation at 95 °C for 1 min followed by annealing at 55 °C for 30 s and repeat denaturation at 95 °C for 30 s. Relative transcript abundance of the amplified miRNA was measured following the ΔΔCt method (Supplementary Table 4) of calculation as mentioned above in the TLDA experiment.

### Expression analysis of select target genes by qPCR

To examine whether altered miRNAs were associated with changes in the expression of predicted target genes, we randomly selected seven genes that show putative binding sites for certain significantly altered miRNAs and have relevance in depression pathophysiology. mRNA levels of these genes were determined by qPCR using EvaGreen/SybrGreen-based reaction chemistry (EvaGreen qPCR Mastermix) as discussed above. The primer sequences for each gene were designed and are as follows: CREB1 (forward: 5′-AGTGGCAGTGCTTAAAAACCAAA-3′; reverse: 5′-CTGACTTGTGGCAGTAAAGGTC-3′), BDNF (forward: 5′-CCCCATCACAATCTCACGGT-3′ reverse: 5′-GTTGCGGAGGGTCTCCTATG-3′), CaMKIIα (forward: 5′-AGACACCAAAGTGCGCAAAC-3′; reverse: 5′-TTCCAGGGTCGCACATCTTC-3′), AKT3 (forward: 5′-TCCCCCGAACACTCTCTTCA-3′ reverse: 5′-CCCTCCACCAAGGCGTTTAT-3′) and NR3C1 (forward: 5′-AAGACTTGGCGACAGAAGCA-3′; reverse: 5′-CCATGCCTCCACGTAACTGT-3′). All the values were normalized using rat-specific GAPDH (forward: 5′CACTGAGCATCTCCCTCACAA-3′, reverse: 5′-TGGTATTCGAGAGAAGGGAGG-3′) as endogenous control. The results were calculated using ΔΔCt method (Supplementary Table 5) and reported as fold change.

## Results

### Effect of chronic CORT administration on body weight gain, adrenal weight and serum CORT level

**Figure 1:**
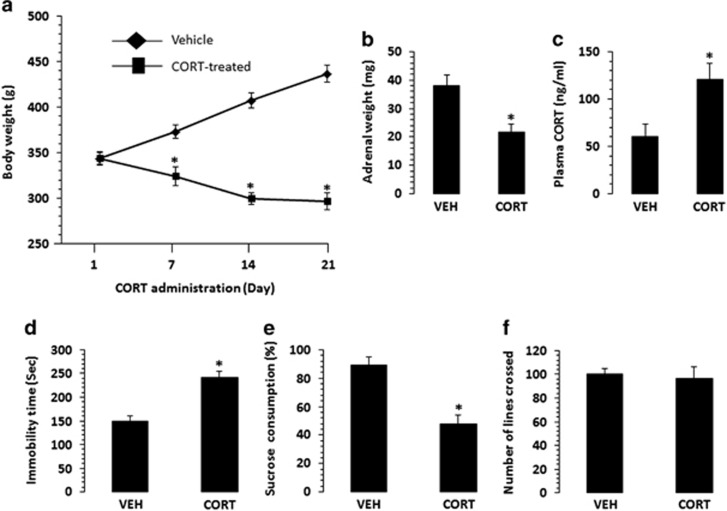
Effects of corticosterone chronic (CORT) administration on various metabolic and behavioral measures. Effects of CORT administration body weight (**a**), adrenal weight (**b**), plasma CORT level (**c**), forced swim test (**d**), sucrose preference test (**e**) and open-field test (**f**). Rats were administered CORT (40 mg  kg^−1^, s.c.) or vehicle (VEH) once daily for 21 days. Weight of rats was taken on day 1, 14 and 21. Adrenal weight and plasma CORT were measured on day 22. Behavioral studies were done on day 22. Values are the means±s.d. (*n*=8 per group). *P<0.001 compared with the vehicle-treated group.

The body weight of the rats was taken on days 1, 7, 14 and 21 and is depicted in Figure 1a. Repeated measures analysis revealed a significant effect of both time (time: *F*_1,14_=75, *P*<0.001) on body weight and interaction between treatment and weight (*F*_1,14_=604, *P*<0.0001). *Post hoc t*-tests further revealed that the groups had similar body weights on day 1 of the injections (*t*_14_=0.04, *P*=0.97), however, after 7 days of injections, the CORT-injected rats weighed significantly less than the vehicle-injected rats (*t*_14_=11.9, *P*<0.001). This effect persisted on days 14 (*t*_14_=29.0, *P*<0.001) and 21 (*t*_14_=30.3, *P*<0.001) of CORT injection. The weight of the adrenal glands was also lower in the CORT-treated group compared with the vehicle-treated group (*t*_14_=9.2, *P*<0.001; Figure 1b).

The serum CORT level is shown in Figure 1c. It was found that the level of CORT was significantly higher in CORT-treated group as compared with the vehicle-treated group (*t*_14_=−9.1, *P*<0.001).

### Effect of chronic CORT administration on forced swim, sucrose preference and open-field tests

The effect of CORT treatment on the immobility time in the FST is given in Figure 1d. As can be seen, CORT-treated rats showed significantly higher immobility time as compared with vehicle-treated rats (*t*_14_=−17.5, *P*=<0.001). Chronic CORT injections in rats also resulted in a significant reduction in the percentage of sucrose consumption compared with the vehicle control rats (*t*_14_=−13.65, *P⩽*0.001; Figure 1e). On the other hand there was no significant difference between chronic CORT-treated and vehicle-treated groups in total lines crossed during the 5-min open-field test (*t*_14_=0.87, *P*=0.40; Figure 1f).

### Effect of chronic CORT administration on miRNA expression in the PFC

As mentioned in the Materials and methods section, miRNAs were assayed on two plates, A and B; A plates contained many of the canonical miRNA sequences in miRBase, whereas the B plate primarily contained minor or * miRNA sequences arising from the opposite arm of the pre-miR hairpin precursor. A total of 734 miRNAs were examined (Supplementary Table 2). Of them, 373 were unique miRNAs for rats. We examined only those miRNAs that showed Ct value ⩽35 as miRNAs showing >35 Ct values (Supplementary Table 2) showed much less reliability when samples were run on duplicate plates. As shown in Table 1, we found that a total of 21 miRNAs were significantly altered in plate A, whereas only 5 miRNAs showed significant change in Plate B. Of 21 miRNAs of plate A, 17 miRNAs were significantly upregulated and 4 were significantly downregulated in the PFC of CORT-treated rats. On the other hand, in plate B, three miRNAs were significantly downregulated and two miRNAs were significantly upregulated (Table 1).

The updated accession numbers, chromosomal coordinates, seed sequences and transcriptional units for each miRNA were obtained from miRbase (v.21) (Manchester, UK) and are shown in Table 1. Out of 26, 16 significantly affected miRNAs were found to be localized at the adjacent genomic loci. For example, miR-218, miR-324-5p, miR-365 and miR-146a were localized on chromosome 10; miR-764-5p and miR-351 on chromosome X; miR-101 and miR-30e on chromosome 5; miR-582 and miR-137 on chromosome 2; miR-153 and miR-203 on chromosome 6; miR-124 and 181a on chromosome 3 and miR-135a*/miR-135a-3p and let-7i on chromosome 7. Some of the miRNAs that were localized on the chromosome and in close proximity showed the same direction of changes. For example, miR-324-5p, miR-365 localized on chromosome 10 and miR-153 and miR-203 localized on chromosome 6 showed significant upregulation. On the other hand, miR-764-5p and miR-351, which are localized on chromosome X showed significant downregulation. The degree of changes for miRNAs localized on the same chromosome was almost the same.

When the promoter regions of altered miRNAs were analyzed (Transfac database v.7.0, Biobase-Qiagen, Waltham, MA, USA), we found that majority of the miRNAs that were modulated by CORT had binding motifs for GR, which were either simple, composite or tethering type within the 1-kb upstream of the transcription start site (Supplementary Figure 1). When most significantly upregulated or downregulated miRNAs (for example, miR-124, miR-218, miR-146a and miR-155) were further analyzed, we found that these miRNAs had at least three simple GR elements (Supplementary Figure 1).

### Validation of select miRNAs by qPCR

To replicate the findings obtained using TLDA array plate, we selected three miRNAs that were upregulated (mir-124, miR-218, miR-29a) and three, miRNAs that were downregulated (miR-146a, miR-200c, miR-155) based on their highest degree of significance by chronic CORT treatment and re-analyzed their expression individually by qPCR. RNA expression of U6 gene was used as a normalizer which did not show significant difference across the two groups (*P*=0.26). The results showed that the relative miRNA levels measured by qPCR were essentially the same as measured by TLDA array plate (Supplementary Figure 2a) and were positively correlated with the fold change found in the TLDA array plate assay (*r*^*2*^=0.98; *P*<0.001; Supplementary Figure 2b).

### Functional analysis of CORT-mediated miRNAs by global and integrated analysis of the miRNA and mRNA expression profile

To further investigate the global association between transcript abundance of miRNAs and their target genes in CORT-associated psychopathology, we analyzed all the 26 miRNAs that were differentially regulated by chronic CORT administration. For this, we used TarBase, Ingenuity Expert Finding and miRecords with experimental validation. In addition, the number of conserved targeting site and total context score prediction value with a high-to-moderate degree of 3′ untranslated region-binding specificity with the miRNA seed sequence were also used. We found that not only a large number of genes were targets of the affected miRNAs (Supplementary Table 6) but altered miRNAs and target gene interaction (selected from Supplementary Table 6) showed a very dense molecular network (Supplementary Figure 3). We confirmed our findings with another software miRWalk v.2 (Mannheim, Germany), which takes into account of miRNA binding sites within the complete sequence of a gene and combines this information with a comparison of binding sites resulting from eight miRNA-target prediction programs (DIANA-microT v4.0 (Athens, Greece), DIANA-microT-CDS (Athens, Greece), miRanda release 2010 (New York, NY, USA), miRDB v.4.0 (St. Louis, MO, USA), PicTar4 (NY, USA and Max Delbruck Centrum, Berlin, Germany), PicTar5 (NY, USA and Max Delbruck Centrum, Berlin, Germany) PITA (Rehovot, Israel), RNA22 v.2 (Philadelphia, PA, USA), RNAhybrid v.2.1 (Bielefeld, Germany) and Targetscan v.6.2).

We found that several highly predicted or experimentally validated target genes had binding sites for multiple miRNAs (Supplementary Table 7). These include: AKT1 (miR-101a, miR-124, miR-181c, 29a, 365), BCL2 (153, 30e, 365), BDNF (124, 30e, 365), CREB (101a, 124, 721, 181c, 203, 218, 582-5p, 351, 155, 200c), DNMT3A (101a, 29a, 30e), ETS (351, 155, 200c), GABAR1 (101a, 721, 137, 181c, 155, 203), GRIA4 (124, 137, 218), GSK3B (155, 101a, 124, 137, 19b, 218, 29a), MAPK1 (miR-101a, 124, 721, 181c, 365), NR3C1(29a, 30e, 365, 582-5p, 124), phosphodiesterase 4 (PDE4a) (101A, 124, 137, 19B) and PDE4D (101a, 124, 721, 137, 30e, 365).

**Figure 2:**
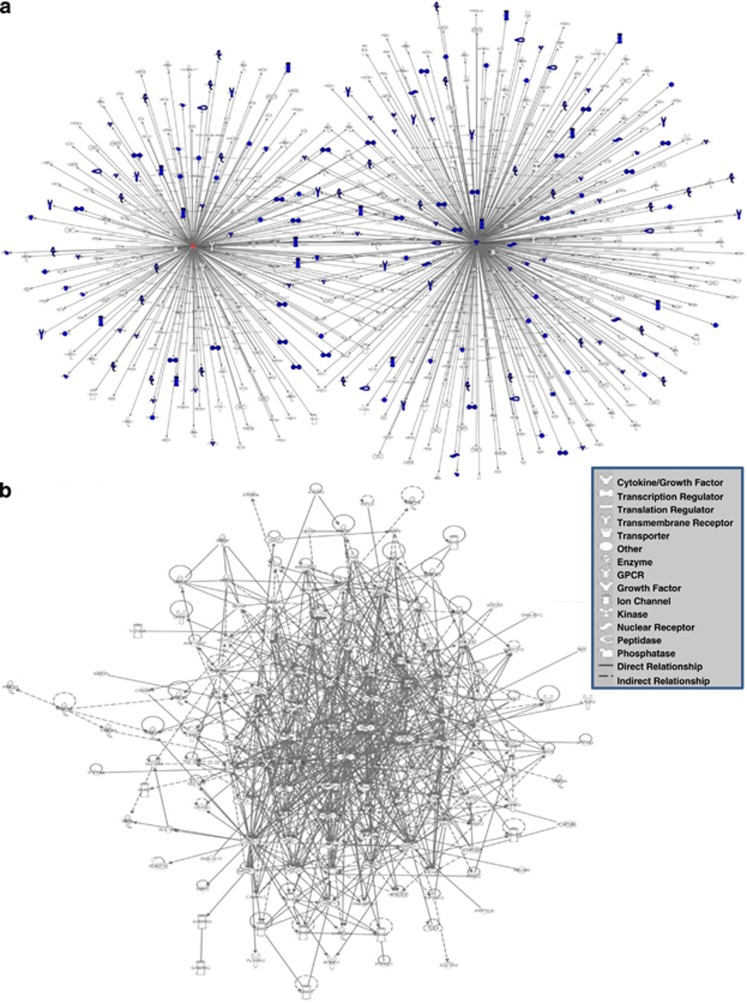
(**a**) miR-218 and 124 with their overlapping targets. Two CORT-mediated significantly altered miRNAs (miR-218 and 124) share multiple target genes. Genes with a role in psychiatric disorder are highlighted in blue. (**b**) Enriched functional network of interconnected molecules as integral part of psychiatric disorders found to be targeted by CORT-induced altered miR-218 and miR-124. The intense molecular crosstalk (represented as solid lines for direct relationship) is indicative of target enrichment of the two highly CORT-mediated upregulated miRNAs (shapes of individual molecules are representative of their function) with profound effect on pathways related to psychiatric disorders. In functional molecular network analysis, genes are represented as nodes. CORT, corticosterone; miRNA, microRNA.

We next selected two specific miRNAs—miR-124 and miR-218—which showed the most statistically significant changes in the CORT-treated group. Target analysis revealed a large number of genes that were affected by these two miRNAs (Figure 2a). Genes that were directly associated with psychological/psychiatric disorders are indicated by blue color. Interestingly, several of these genes (CREB1, MECP2, GRIA2, GRIA4, SP1, PIK3C2A, NFATC1, GSK3β) showed an overlapping pattern of regulation by these two miRNAs. Further functional network analysis based on regulatory relationship between miR-124 and miR-218 target genes showed critical genes that have earlier been reported to be the stress-related pathology (Figure 2b). These include: BDNF, GRs (NR3C1 and NR3C2), CREB1, glutamate receptors NMDA1 and NMDA3 (GRIN1, GRIN3), PDE4A, PDE4B, vesicular monoamine transporter (SLC18A2), glutamate receptors AMPA2 (GRIA2), AMPA3 (GRIA3), AMPA4 (GRIA4), protein tyrosine phosphatase (PTPN1), MARCKS, BCl2, STAT3, SMAD4, IL6, IL10, GABA, glutamate, GABA or dopamine transporters SLC1A2, SLC6A1, SLC6A3, MEF2C, GSK3β, transcriptional repressor methyl-CpG-binding protein 2 (MECP2), glutamate receptor, ionotropic kainate 2 (GRIK2) and HDAC5 and HDAC9. As indicated in Figure 2b, these genes either had direct regulatory relationship with each other or were indirectly related.

**Figure 3:**
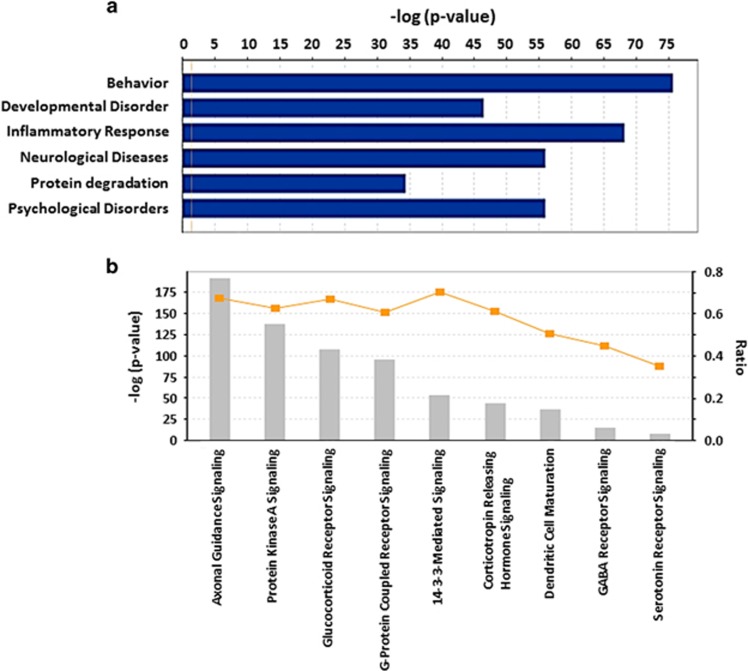
(**a**) Disordered pathways affected by altered miRNAs. Various categories of disorders or disease pathways associated with genes that are predicted to be targets of significantly altered miRNAs in the CORT-treated group are shown (*P*<0.05, Fisher's Exact Test). (**b**) Canonical pathways affected by CORT-mediated altered miRNAs. Canonical biological pathways associated with genes that are predicted to be targets of CORT-mediated significantly altered miRNAs is shown (*P*<0.05, Fisher's Exact Test). The ratio is calculated as the number of genes in a given pathway divided by the number of genes that make up the pathway. The *P*-value for a given process annotation is calculated by considering the number of focus genes that participate in that process and the total number of genes that are known to be associated with that process in the selected reference set. The more focus genes involved, the more likely the association is not due to a random chance. CORT, corticosterone; miRNA, microRNA.

**Figure 4:**
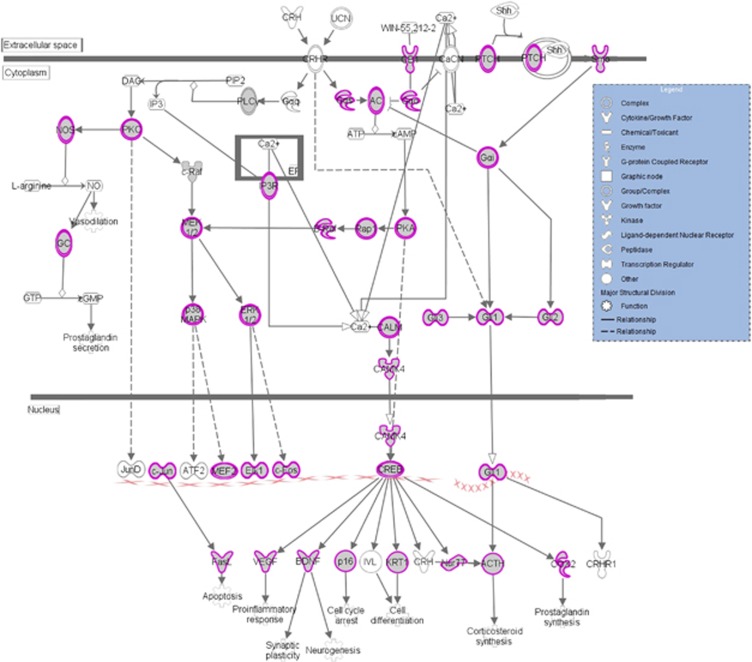
Elaboration of one of the canonical pathways, corticotropin-releasing hormone (CRH), affected by altered miRNAs in the CORT-treated group. Genes encircled in the pink color denote targets of altered miRNAs. IPA software was used to generate this pathway. IPA, Ingenuity Pathway Analysis Software; miRNA, microRNA.

To examine the phenotypes associated with CORT-induced altered miRNAs, we performed mapping of genes to known human diseases and disorders. The top five disorders included developmental, inflammatory, neurological, psychological and protein degradation (Figure 3a). Among psychological disorders, major depressive disorder was associated with 88 predicated genes, whereas overall mood disorder was associated with 133 predicted genes. Further canonical pathways analysis (Fisher Exact Test and *P*-value threshold set at 0.05) revealed nine functionally relevant pathways that were of high interest (Figure 3b). Of them, the axonal guidance pathway contained the most affected genes (266 genes) followed by protein kinase A (207 genes) and GR (154 genes) signaling. In addition, corticotropin-releasing hormone signaling (68 genes), dendritic cell maturation (69 genes) and GABA receptor signaling (30 genes) were of high interest. A number of serotonergic genes (18 genes) also appeared in the list that was predicated to be affected by multiple miRNAs. We further analyzed corticotropin-releasing hormone signaling pathway in detail and found that several genes associated with stress signaling are directly affected by CORT-induced altered miRNAs. As shown in Figure 4, some of the these genes included: *PKC*, *MEK1/2*, *p38 MAPK*, *ERK1/2*, *Rap1*, *PKA*, *CaMK4*, *c-Jun*, *Fas-L*, *BDNF* and *CREB*, which can lead to altered pro-inflammatory response, neurogenesis, cell differentiation, corticosteroid synthesis and cell survival.

### mRNA analyses of select target genes in PFC of CORT-treated rats

The relative transcript abundance of predicted target genes for a few significantly upregulated miRNAs were analyzed in PFC of vehicle and CORT-treated rats by qPCR. GAPDH was used as endogenous control, which did not differ between vehicle and CORT-treated groups (*P*=0.21). As can be seen in Supplementary Figure 4, we found that expression of CREB (*P*<0.001), BDNF (*P*=0.012), CaMKIIα (*P*=0.002), AKT3 (0.044) and NR3C1 (*P*=0.014) were significantly decreased in CORT-treated group. We did not find any significant difference in the expression of PTEN (*P*=0.48) and VEGFA (*P*=0.72) genes in CORT-treated rats, although there was a trend of decrease in both these genes.

## Discussion

Chronic CORT-induced depression in rodents is a well-established animal model that allows examination of the direct effects of glucocorticoids on the development of depressive symptomatology.^20^ In this model, rodents show not only profound maladaptive changes in emotional behavior and dysregulated HPA axis functions but also key features that resemble phenotypic characteristics of clinical depression. These include FST immobility, decreased sucrose preference, decreased sexual and grooming behavior, decreased reward behavior, and impaired spatial working memory and executive functions.^20, 29^ Many of these behavioral changes are reversed by antidepressant treatment^30^ supporting the predictive validity of this model. In the present study, we found that chronic CORT-treated animals showed significantly lower body weight gain over a period of 21 days, as well as lower adrenal weight, which are the two key physiological manifestations of depression that have earlier been reported in chronic CORT-treated animals.^20, 22, 24, 31, 32^ As have been shown in these studies as well as in our present study, the depression-like behavior in CORT-treated animals are not due to body weight loss as these rats do not show changes in locomotors, open field or Morris Water maze test.^21, 22, 33^ In fact, this effect has been attributed to the catabolic effects of CORT on muscle tissue.^32, 34^ Alternatively, stress-induced changes in energy expenditure may be part of weight loss since there is no change in food intake between vehicle and CORT-treated rats.^35, 36^ To assess depression-like behavior, we examined FST and sucrose preference test and found that chronic CORT-treated rats spent a significantly greater percentage of time immobile and showed decreased sucrose preference. When locomotors activity was examined, CORT-treated rats did not show any change in the open field. Although not assessed in the present study, in the Morris Water maze, rats subjected to repeated high doses of CORT show similar swim distances to control rats.^37^ All these evidences collectively suggest that depression-like behavior in these animals is not associated with nonspecific motor behavior. These findings are also quite similar to those reported previously.^22, 23, 24, 29, 38^

We examined miRNA expression in PFC for several reasons: (1) PFC is a major target for glucocorticoids and mediates many of the behaviors influenced by stress.^39, 40^ This brain area is directly targeted by stress hormones via GRs and is implicated in feed-back control of HPA axis activity;^41, 42^ (2) several studies show that pathological activation of prefrontal cortical GR by chronic stress negatively impacts GR expression and causes dendritic atrophy and spine loss, suggesting both a loss of prefrontal feed-back control and altered neuronal excitability;^39, 43^ (3) PFC shows morphological changes after chronic CORT administration similar to those observed in depressed patients;^39, 44^ and importantly (4) significant alterations in expression of miRNAs have been noted in PFC of depressed individuals^17^ and learned helpless rats.^18^

In the present study, we found that chronic CORT administration to rats caused altered expression of 26 miRNAs in the PFC. Of them 19 were upregulated (let-7i, miR-19b, miR-29c, miR-101a, miR-124, miR-137, miR-153, miR-181a, miR-181c, miR-203, miR-218, miR-324-5p, miR-365, miR-409-5p, miR-582-5p, miR-155, miR-29a, miR-30e, miR-721, miR-699) and 7 were downregulated (miR-146a, miR-200c, miR-351, miR-155, miR-678, miR-764-5p, miR-135a*). We confirmed this finding by analyzing the six most significant CORT-induced altered miRNAs (miR-218, miR-124, miR-29a, miR-146a, miR-200c, miR-155) by qPCR. These changes in miRNA expression level in frontal cortex reflect the responsiveness to hyperactive HPA axis as direct representation of increased serum cortisol level in CORT-treated rats, which could be relevant in the development of depressive phenotype,^45, 46^ although further studies will be needed to show their direct relationship.

*In silico* mapping of genes associated with CORT-induced altered miRNAs showed that a large number of genes were associated with developmental, inflammatory and psychological disorder phenotypes. This is not surprising given the role of CORT in abnormal brain development,^47^ inflammatory response,^48^ and mood and anxiety disorders.^20^ In this respect, target gene analysis of individual altered miRNAs revealed a large number of genes that have been shown to be associated with inflammation, synaptic plasticity, cell differentiation, cell survival, cell adhesion and epigenetic modifications. For example, several CORT-mediated, altered miRNAs showed target recognition sites in the inflammation-related genes such as interleukin (IL) 1A, IL2, IL6, IL10, IL10R, IL6R, TLR1, TLR4, TLR9, TLR10, TNF, TNFAIP6 and TNFAIP3. Some of these pro-inflammatory cytokines, TNF-α as well as Toll-like receptors, have been implicated in glucocorticoid-mediated stress response^49^ and in the etiology of major depression.^50^ MiR-101a, miR-124, miR-721, miR-181c and miR-365 target ERK/MAPK1, a gene involved in several physiological functions in brain including cell proliferation, differentiation and cell survival. MAPK1 has not only been shown to be downregulated in PFC of depressed subjects^51, 52^ and in the frontal cortex of chronic restraint rats^53^ but its downregulation can lead to depressive phenotype in rodents.^54^ Phospholipase D, whose roles in cell communication and a wide range of biological processes are well demonstrated and whose expression is compromised in rat model of depression,^55^ is the target of miR-324-5p. CaMKII, a target gene for miR-137 and miR-324-5p, is well-known for its role in synaptic plasticity.^56^ This gene has also been shown to be less expressed in frontal cortex of depressed patients.^57^ Another synaptic plasticity-related gene CREB is a target of multiple miRNAs (miR-101a, miR-203, miR-218, miR-721, miR-409-5p). Interestingly its target gene BDNF, whose expression is compromised in depressed brain^58^ and whose expression and functions are regulated by CORT,^59^ is predicted to be regulated by miR-124 and miR-30e. It is pertinent to mention that CREB itself is less expressed in PFC of depressed individuals.^60^ Several axonal guidance, cytoskeleton and cell adhesion genes (ABL1, EPHA10, NFATC1, PLCB3, ACTC1) that are critical for structural plasticity^61^ are predicted to be targeted by miR-351 and miR-146a. Several components of PI3 kinase signaling such as AKT3, PTEN, PIK3C2A and PIK3C2, which play critical roles in neurotrophin-mediated signaling and cell survival,^62^ are targets of miR-29a, miR-101a, miR-124, miR-181c and miR-678. Earlier studies have shown that PIK3C2A and PIK3C2B are downregulated and PTEN is upregulated in the PFC of depressed subjects.^63^ A large number of cyclic AMP-specific PDEs are also targets of multiple miRNAs (miR-101a, miR-124, miR-721, miR-137, miR-19b, miR-30e, miR-365). These PDEs have not only been associated with depression but they possess antidepressants and cognitive-enhancing effects.^64^ Serotonergic genes HTR2A and HTR2C, and SLC6A4 (5HTTLPR) are targeted by miR-203 and miR-324-5p, respectively. HTR2A and HTR2C are critical for HPA axis dysregulation, depression and anxiety^65^ whereas SLC6A4 polymorphism contributes to depression and familial transmission of vulnerability to emotionality.^66^ Epigenetic associated genes DNMT3A and DNMT 3B, targets of miR-101a, 124, 29a, 30e, have been found to be upregulated in the PFC of depressed suicide subjects.^67^ On the other hand, MECP2, a transcriptional regulator targeted by miR-19, miR-30e and miR-365, binds to methylated DNA and has been shown to contribute to early life stress-dependent epigenetic programming of HPA axis-associated genes.^68^ Interestingly, when examined, we found that expression levels of CREB1, BDNF, CAMKIIa, AKT1, and NR3C1 were significantly decreased in PFC of CORT-treated rats. As listed in Supplementary Table 6, these genes were predicted targets of miR-124, miR-101, miR-29a, miR-30e, miR-181c, miR-365 and miR-218. These miRNAs were upregulated in CORT-treated group, suggesting an inverse correlation between these miRNAs and their target genes.

Our analysis showed that some of the miRNAs whose chromosomal localizations were in close proximity had the same direction of changes. In addition, we found that several genes were predicted to be targeted by the same miRNA. These notions signify the evolutionary conservation pattern of gene regulation which may culminate into similar functional output.^69^ This may also have relevance where CORT may be regulating functional gene networks in a cohesive manner in inducing distinct depression phenotype. In an earlier study, we reported that normal homeostatic miRNA response to repeated inescapable shock was not merely blunted in frontal cortex of learned helpless rats compared with non-helpless rats but gene expression networks were actively reorganized, giving unique phenotypic learned helpless characteristics to these rats.^18^

The precise molecular mechanisms by which CORT regulates miRNAs are not clear; however, we found that all the altered miRNAs possessed putative binding sites for GR. Interestingly, the most significantly altered miRNAs had at least three simple GR elements in the upstream promoter element, suggesting that there is a direct interaction of GR homodimer with these binding sites, which could lead to altered expression of these miRNAs. Besides, we found a few GR elements that were composite or tethering in nature. This could lead to a fine tuning of miRNA regulation in conjunction with other transcription factors. It appears that CORT may be altering transcription of miRNAs by regulating either binding of transcription factors to their putative binding sites or recruitment of transcription factors to specific response elements.

In this study, we found that miR-124 was the most significantly upregulated miRNA in the PFC of CORT-treated rats. miR-124 is specifically expressed in brain^70^ and has a significant role in maintaining neuronal cell identity^71^ and synaptic plasticity.^72^ Earlier, Vreugdenhil *et al.*^73^ showed that miRNA-124 binds to the 3′ untranslated region of GR (NR3C1) and decreases its activity. Interestingly, these investigators also found that physiological miRNA-124 expression levels in several brain areas exceeds that of what is needed to reduce GR protein levels, suggesting a critical role for miRNA-124 in regulating GR activity. Since expression of NR3C1 gene is reduced after CORT administration,^74^ our finding of upregulated miR-124 suggests that decreased GR by CORT could be associated with increased expression of miR-124. Not only GR but mineralocorticoid receptor is also a target of miR-124. It has been shown that acute stress regulates miR-124 in amygdala of mice in a negative manner which is inversely correlated with mineralocorticoid receptor expression.^71^ As with our present study, Cao *et al.*^75^ reported upregulation of miR-124 in hippocampus of rats subjected to unpredictable chronic mild stress, another model of depression. Interestingly, in Aplysia, serotonin rapidly and robustly regulates miRNA-124.^72^ MiR-124 responds to serotonin by de-repressing CREB and thereby enhances serotonin-dependent long-term facilitation.^72^ In future, it will be interesting to examine whether manipulation of miR-124 can lead to depression phenotype.

Our study also shows that one of the highly predictable canonical pathways that are affected by CORT is corticotropin-releasing hormone signaling. Individual target analysis suggests that chaperone proteins FKBP4 and FKBP5 that are critical for the binding affinity of GR and consequent corticotropin-releasing hormone-mediated stress response are targets of a set of miRNAs: miR-203, 721, 29a, and 137. FKBP5 has not only emerged as a strong candidate gene in depression^76^ but genetic variation in FKBP5 has been shown to be associated with the extent of stress hormone dysregulation in this disorder.^77^ FKBP5 gene is also altered by chronic exposure to stress and knocking down this gene shapes the behavioral and neuroendocrine phenotype in rodents.^78^

Altogether, our study points to probable mechanisms by which chronic CORT may induce depression phenotype by altering a select group of miRNAs and associated networks. This inference needs to be further supported by additional *in vivo* experiments. Recently, Rinaldi *et al.*^79^ reported that acute restraint stress causes a transient increase in the expression of selected miRNAs in the frontal cortex. It will be interesting to examine how these miRNAs are regulated under acute CORT response as maladaptive response to long-term elevated CORT is responsible for inducing depression. Delineating acute vs chronic CORT responses will thus differentiate adaptive and maladaptive miRNA-induced network changes and their role in the development of depression phenotype. In addition, it will be critical to correlate our findings with mRNA and protein expression of target genes.

## Figures and Tables

**Table 1:** CORT-mediated (50 mg  kg^−1^, 21 days s.c.), altered miRNAs in rat prefrontal cortex and their characteristics

*miRNAs*	*Accession no.*	*Fold change*	P*-value*	*Regulation*	*Chromosomal location (Rat)*	*Seed sequence*	*Transcriptional unit*
miR-19b	MIMAT0000788	1.284078291	0.015148	↑	chr15: 103641487-103641573 [+] chrX: 140167226-140167321 [−]	5′-GUGCAAA-3′	Intergenic, intergenic
miR-29c	MIMAT0000803	1.565815431	0.004636	↑	chr1: 39612916-39613003 [+] chr13: 118329978-118330065 [+]	5′-AGCACCA-3′	Exonic, exonic
miR-101a	MIMAT0000823	1.594450158	0.028947	↑	chr5: 124050126-124050200 [−]	5′-ACAGUAC-3′	Exonic
miR-124	MIMAT0000828	1.768260098	0.000853	↑	chr3: 180116126-180116212 [+] chr15: 51712751-51712835 [+] chr2: 122049028-122049136 [+]	5′-AAGGCAC-3′	Intergenic, intergenic, intergenic
miR-137	MIMAT0000843	1.394684534	0.009357	↑	chr2: 239707763-239707864 [+]	5′-UAUUGCU-3′	Intergenic
miR-153	MIMAT0000855	2.328177683	0.015395	↑	chr6: 154028732-154028818 [+]	5′-UGCAUAG-3′	Intronic
miR-181a	MIMAT0000858	1.443247107	0.034332	↑	chr3: 28374769-28374885 [+] chr13: 59986075-59986174 [+]	5′-ACAUUCA-3′	Intronic, exonic
miR-181c	MIMAT0000857	1.731680672	0.012072	↑	chr19: 36267311-36267416 [−]	5′-ACAUUCA-3′	Intergenic
miR-203	MIMAT0000876	1.39812046	0.033212	↑	chr6: 145714633-145714729 [+]	5′-UGAAAUG-3′	Exonic
miR-218	MIMAT0000888	1.285324379	0.000235	↑	chr10: 20303158-20303267 [+] chr14: 66955494-66955603 [−]	5′-UGUGCUU-3′	Intronic, exonic, intronic
miR-324-5p	MIMAT0000553	1.412569727	0.049794	↑	chr10: 56366245-56366327 [+]	5′-GCAUCCC-3′	Exonic, intronic
miR-365	MIMAT0001549	1.279026214	0.035465	↑	chr10: 64570072-64570157 [−]	5′-AAUGCCC-3′	Intergenic
miR-409-5p	MIMAT0003204	1.497710118	0.047871	↑	chr6: 143055776-143055852 [+]	5′-GGUUACC-3′	Exonic
miR-582-5p	MIMAT0012833	1.810050564	0.008875	↑	chr2: 59928607-59928687 [+]	5′-UACAGUU-3′	Intronic
miR-29a	MIMAT0000802	1.594299491	0.003691	↑	chr4: 58099674-58099761 [−]	5′-AGCACCA-3′	Exonic
miR-30e	MIMAT0000805	1.380416302	0.02323	↑	chr5: 143497752-143497843 [−]	5′-GUAAACA-3′	Intronic
miR-721	No report	1.691847218	0.037392	↑	Not reported	5′-AGUGCAA-3′	Not reported
miR-699	No report	2.007193854	0.008792	↑	Not reported	Not reported	Not reported
miR-146a	MIMAT0000852	0.603748944	0.006804	↓	chr10: 28806739-28806833 [−]	5′-GAGAACU-3′	Intergenic
miR-200c	MIMAT0000873	0.114793637	0.016703	↓	chr4: 224254382-224254450 [−]	5′-AAUACUG-3′	Exonic
miR-351	MIMAT0000608	0.654310843	0.025434	↓	chrX: 152774513-152774593 [+] chrX: 153212230-153212310 [+]	5′-CCCUGAG-3′	Intergenic, intergenic
miR-155	MIMAT0030409	0.526008585	0.020242	↓	chr11: 27810358-27810422 [+]	5′-UAAUGCU-3′	Intergenic
miR-678	MIMAT0012857	0.679476805	0.038127	↓	chr20: 15366762-15366844 [+]	5′-UCUCGGG-3′	Exonic
miR-764-5p	MIMAT0012854	0.66665086	0.032063	↓	chrX: 118264668-118264775 [+]	5′-GUGCUCA-3′	Intergenic
miR-135a-3p	MIMAT0004732	0.738454042	0.02441	↓	chr7: 32974076-32974175 [−]	5′-AUGGCUU-3′	Intergenic

Abbreviations: CORT, corticosterone; miR, microRNA.

## References

[bib1] Bromet E, Andrade LH, Hwang I, Sampson NA, Alonso J, de Girolamo G et al. Cross-national epidemiology of DSM-IV major depressive episode. BMC Med 2011; 9: 90. 21791035 10.1186/1741-7015-9-90 PMC3163615

[bib2] Möller HJ. Suicide, suicidality and suicide prevention in affective disorders. Acta Psychiatr Scand Suppl 2003; 418: 73–80. 12956819

[bib3] Warden D, Rush AJ, Trivedi MH, Fava M, Wisniewski SR. The STAR*D Project results: a comprehensive review of findings. Curr Psychiatry Rep 2007; 9: 449–459. 18221624 10.1007/s11920-007-0061-3

[bib4] Labermaier C, Masana M, Müller MB. Biomarkers predicting antidepressant treatment response: how can we advance the field? Dis Markers 2013; 35: 23–31. 24167346 10.1155/2013/984845 PMC3774965

[bib5] Peña CJ, Bagot RC, Labonté B, Nestler EJ. Epigenetic signaling in psychiatric disorders. J Mol Biol 2014; 426: 3389–3412. 24709417 10.1016/j.jmb.2014.03.016 PMC4177298

[bib6] Gold PW. The organization of the stress system and its dysregulation in depressive illness. Mol Psychiatry 2015; 20: 32–47. 25486982 10.1038/mp.2014.163

[bib7] Caputo V, Ciolfi A, Macrì S, Pizzuti A. The emerging role of microrna in schizophrenia. CNS Neurol Disord Drug Targets 2015; 14: 208–221. 25613509 10.2174/1871527314666150116124253

[bib8] Kocerha J, Dwivedi Y, Bernnand K. Non-coding RNAs and neurobehavioral mechanisms in psychiatric disease. Mol Psychiatry 2015; 20: 677–684. 25824307 10.1038/mp.2015.30 PMC4440836

[bib9] Wilczynska A, Bushell M. The complexity of miRNA-mediated repression. Cell Death Differ 2015; 22: 22–33. 25190144 10.1038/cdd.2014.112 PMC4262769

[bib10] Dwivedi Y. Evidence demonstrating role of microRNAs in the etiopathology of major depression. J Chem Neuroanat 2011; 42: 142–156. 21515361 10.1016/j.jchemneu.2011.04.002 PMC3163781

[bib11] Im HI, Kenny PJ. MicroRNAs in neuronal function and dysfunction. Trends Neurosci 2012; 35: 325–334. 22436491 10.1016/j.tins.2012.01.004 PMC3565236

[bib12] Jiang P, Coller H. Functional interactions between microRNAs and RNA binding proteins. MicroRNA 2012; 1: 70–79. 25048093 10.2174/2211536611201010070 PMC5123774

[bib13] Malphettes L, Fussenegger M. Impact of RNA interference on gene networks. Metab Eng 2006; 8: 672–683. 16996764 10.1016/j.ymben.2006.07.005

[bib14] Garbett KA, Vereczkei A, Kálmán S, Brown JA, Taylor WD, Faludi G et al. Coordinated messenger RNA/microRNA changes in fibroblasts of patients with major depression. Biol Psychiatry 2015; 77: 256–265. 25016317 10.1016/j.biopsych.2014.05.015 PMC4254393

[bib15] Issler O, Haramati S, Paul ED, Maeno H, Navon I, Zwang R et al. MicroRNA 135 is essential for chronic stress resiliency, antidepressant efficacy, and intact serotonergic activity. Neuron 2014; 83: 344–360. 24952960 10.1016/j.neuron.2014.05.042

[bib16] Lopez JP, Lim R, Cruceanu C, Crapper L, Fasano C, Labonte B et al. miR-1202 is a primate-specific and brain-enriched microRNA involved in major depression and antidepressant treatment. Nat Med 2014; 20: 764–768. 24908571 10.1038/nm.3582 PMC4087015

[bib17] Smalheiser NR, Lugli G, Rizavi HS, Torvik VI, Turecki G, Dwivedi Y. MicroRNA expression is down-regulated and reorganized in prefrontal cortex of depressed suicide subjects. PLoS One 2012; 7: e33201. 22427989 10.1371/journal.pone.0033201 PMC3302855

[bib18] Smalheiser NR, Lugli G, Rizavi HS, Zhang H, Torvik VI, Pandey GN et al. MicroRNA expression in rat brain exposed to repeated inescapable shock: differential alterations in learned helplessness vs. non-learned helplessness. Int J Neuropsychopharmacol 2011; 14: 1315–1325. 21275079 10.1017/S1461145710001628

[bib19] Smalheiser NR, Zhang H, Dwivedi Y. Enoxacin elevates microRNA levels in rat frontal cortex and prevents learned helplessness. Front Psychiatry 2014; 5: 6. 24575053 10.3389/fpsyt.2014.00006 PMC3918929

[bib20] Sterner EY, Kalynchuk LE. Behavioral and neurobiological consequences of prolonged glucocorticoid exposure in rats: relevance to depression. Prog Neuropsychopharmacol Biol Psychiatry 2010; 34: 777–790. 20226827 10.1016/j.pnpbp.2010.03.005

[bib21] Kalynchuk LE, Gregus A, Boudreau D, Perrot-Sinal TS. Corticosterone increases depression-like behavior, with some effects on predator odor-induced defensive behavior, in male and female rats. Behav Neurosci 2004; 118: 1365–1377. 15598145 10.1037/0735-7044.118.6.1365

[bib22] Gregus A, Wintink AJ, Davis AC, Kalynchuk LE. Effect of repeated corticosterone injections and restraint stress on anxiety and depression-like behavior in male rats. Behav Brain Res 2005; 156: 105–114. 15474655 10.1016/j.bbr.2004.05.013

[bib23] Johnson SA, Fournier NM, Kalynchuk LE. Effect of different doses of corticosterone on depression-like behavior and HPA axis responses to a novel stressor. Behav Brain Res 2006; 168: 280–288. 16386319 10.1016/j.bbr.2005.11.019

[bib24] Marks W, Fournier NM, Kalynchuk LE. Repeated exposure to corticosterone increases depression-like behavior in two different versions of the forced swim test without altering nonspecific locomotor activity or muscle strength. Physiol Behav 2009; 98: 67–72. 19393673 10.1016/j.physbeh.2009.04.014

[bib25] Paxinos G, Watson C. The Rat Brain in Stereotaxic Coordinates, 4th edn, Academic Press: Sydney, NSW, Australia, 1998.

[bib26] Mao QQ, Huang Z, Ip SP, Xian YF, Che CT. Peony glycosides reverse the effects of corticosterone on behavior and brain BDNF expression in rats. Behav Brain Res 2012; 227: 305–309. 22119711 10.1016/j.bbr.2011.11.016

[bib27] O'Donovan S, Kennedy M, Guinan B, O'Mara S, McLoughlin DM. A comparison of brief pulse and ultrabrief pulse electroconvulsive stimulation on rodent brain and behaviour. Prog Neuropsychopharmacol Biol Psychiatry 2012; 37: 147–152. 22230649 10.1016/j.pnpbp.2011.12.012

[bib28] Lugli G, Torvik VI, Larson J, Smalheiser NR. Expression of microRNAs and their precursors in synaptic fractions of adult mouse forebrain. J Neurochem 2008; 106: 650–661. 18410515 10.1111/j.1471-4159.2008.05413.x PMC3711666

[bib29] Lussier AL, Romay-Tallón R, Caruncho HJ, Kalynchuk LE. Altered GABAergic and glutamatergic activity within the rat hippocampus and amygdala in rats subjected to repeated corticosterone administration but not restraint stress. Neuroscience 2013; 231: 38–48. 23206875 10.1016/j.neuroscience.2012.11.037

[bib30] Zahorodna A, Hess G. Imipramine and citalopram reverse corticosterone-induced alterations in the effects of the activation of 5-HT(1A) and 5-HT(2) receptors in rat frontal cortex. J Physiol Pharmacol 2006; 57: 389–399. 17033092

[bib31] Bisagno V, Ferrini M, Ríos H, Zieher LM, Wikinski SI. Chronic corticosterone impairs inhibitory avoidance in rats: possible link with atrophy of hippocampal CA3 neurons. Pharmacol Biochem Behav 2000; 66: 235–240. 10880674 10.1016/s0091-3057(00)00265-3

[bib32] Zhao Y, Ma R, Shen J, Su H, Xing D, Du L. A mouse model of depression induced by repeated corticosterone injections. Eur J Pharmacol 2008; 581: 113–120. 18184609 10.1016/j.ejphar.2007.12.005

[bib33] Brotto LA, Gorzalka BB, Barr AM. Paradoxical effects of chronic corticosterone on forced swim behaviours in aged male and female rats. Eur J Pharmacol 2001; 424: 203–209. 11672564 10.1016/s0014-2999(01)01148-7

[bib34] Quan ZY, Walser M. Effect of corticosterone administration at varying levels on leucine oxidation and whole body protein synthesis and breakdown in adrenalectomized rats. Metabolism 1991; 40: 1263–1267. 1961118 10.1016/0026-0495(91)90026-s

[bib35] Chotiwat C, Harris RB. Antagonism of specific corticotropin-releasing factor receptor subtypes selectively modifies weight loss in restrained rats. Am J Physiol Regul Integr Comp Physiol 2008; 295: R1762–R1773. 18922964 10.1152/ajpregu.00196.2008 PMC2685294

[bib36] Scherer IJ, Holmes PV, Harris RB. The importance of corticosterone in mediating restraint-induced weight loss in rats. Physiol Behav 2011; 102: 225–233. 21092743 10.1016/j.physbeh.2010.11.014 PMC3010503

[bib37] Sousa N, Lukoyanov NV, Madeira MD, Almeida OF, Paula-Barbosa MM. Reorganization of the morphology of hippocampal neurites and synapses after stress-induced damage correlates with behavioral improvement. Neuroscience 2000; 97: 253–266. 10799757 10.1016/s0306-4522(00)00050-6

[bib38] Hill MN, Brotto LA, Lee TT, Gorzalka BB. Corticosterone attenuates the antidepressant-like effects elicited by melatonin in the forced swim test in both male and female rats. Prog Neuropsychopharmacol Biol Psychiatry 2003; 27: 905–911. 14499306 10.1016/S0278-5846(03)00149-0

[bib39] Wellman CL. Dendritic reorganization in pyramidal neurons in medial prefrontal cortex after chronic corticosterone administration. J Neurobiol 2001; 49: 245–253. 11745662 10.1002/neu.1079

[bib40] Mizoguchi K, Ishige A, Aburada M, Tabira T. Chronic stress attenuates glucocorticoid negative feedback: involvement of the prefrontal cortex and hippocampus. Neuroscience 2003; 119: 887–897. 12809708 10.1016/s0306-4522(03)00105-2

[bib41] Diorio D, Viau V, Meaney MJ. The role of the medial prefrontal cortex (cingulate gyrus) in the regulation of hypothalamic-pituitary-adrenal responses to stress. J Neurosci 1993; 13: 3839–3847. 8396170 10.1523/JNEUROSCI.13-09-03839.1993 PMC6576467

[bib42] Radley JJ, Arias CM, Sawchenko PE. Regional differentiation of the medial prefrontal cortex in regulating adaptive responses to acute emotional stress. J Neurosci 2006; 26: 12967–12976. 17167086 10.1523/JNEUROSCI.4297-06.2006 PMC6674963

[bib43] Radley JJ, Rocher AB, Rodriguez A, Ehlenberger DB, Dammann M, McEwen BS et al. Repeated stress alters dendritic spine morphology in the rat medial prefrontal cortex. J Comp Neurol 2008; 507: 1141–1150. 18157834 10.1002/cne.21588 PMC2796421

[bib44] Cerqueira JJ, Pêgo JM, Taipa R, Bessa JM, Almeida OF, Sousa N. Morphological correlates of corticosteroid-induced changes in prefrontal cortex-dependent behaviors. J Neurosci 2005; 25: 7792–7800. 16120780 10.1523/JNEUROSCI.1598-05.2005 PMC6725252

[bib45] Gądek-Michalska A, Spyrka J, Rachwalska P, Tadeusz J, Bugajski J. Influence of chronic stress on brain corticosteroid receptors and HPA axis activity. Pharmacol Rep 2013; 65: 1163–1175. 24399712 10.1016/s1734-1140(13)71474-9

[bib46] McKlveen JM, Myers B, Flak JN, Bundzikova J, Solomon MB, Seroogy KB et al. Role of prefrontal cortex glucocorticoid receptors in stress and emotion. Biol Psychiatry 2013; 74: 672–679. 23683655 10.1016/j.biopsych.2013.03.024 PMC3797253

[bib47] Brown GR, Spencer KA. Steroid hormones, stress and the adolescent brain: a comparative perspective. Neuroscience 2013; 26: 115–128. 10.1016/j.neuroscience.2012.12.016 23262238

[bib48] Silverman MN, Sternberg EM. Glucocorticoid regulation of inflammation and its functional correlates: from HPA axis to glucocorticoid receptor dysfunction. Ann NY Acad Sci 2012; 1261: 55–63. 22823394 10.1111/j.1749-6632.2012.06633.x PMC3572859

[bib49] Gądek-Michalska A, Tadeusz J, Rachwalska P, Bugajski J. Cytokines, prostaglandins and nitric oxide in the regulation of stress-response systems. Pharmacol Rep 2013; 65: 1655–1662. 24553014 10.1016/s1734-1140(13)71527-5

[bib50] Young JJ, Bruno D, Pomara N. A review of the relationship between proinflammatory cytokines and major depressive disorder. J Affect Disord 2014; 169: 15–20. 25128861 10.1016/j.jad.2014.07.032

[bib51] Dwivedi Y, Rizavi HS, Conley RR, Pandey GN. ERK MAP kinase signaling in post-mortem brain of suicide subjects: differential regulation of upstream Raf kinases Raf-1 and B-Raf. Mol Psychiatry 2006; 11: 86–98. 16172610 10.1038/sj.mp.4001744

[bib52] Dwivedi Y, Rizavi HS, Zhang H, Roberts RC, Conley RR, Pandey GN. Aberrant extracellular signal-regulated kinase (ERK)1/2 signalling in suicide brain: role of ERKkinase 1 (MEK1). Int J Neuropsychopharmacol 2009; 12: 1337–1354. 19835659 10.1017/S1461145709990575

[bib53] Leem YH, Yoon SS, Kim YH, Jo SA. Disrupted MEK/ERK signaling in the medial orbital cortex and dorsal endopiriform nuclei of the prefrontal cortex in a chronic restraint stress mouse model of depression. Neurosci Lett 2014; 580: 163–168. 25116759 10.1016/j.neulet.2014.08.001

[bib54] Qi X, Lin W, Wang D, Pan Y, Wang W, Sun M. A role for the extracellular signal-regulated kinase signal pathway in depressive-like behavior. Behav Brain Res 2009; 199: 203–209. 19159647 10.1016/j.bbr.2008.11.051

[bib55] Feng P, Huang C. Phospholipase D-mTOR signaling is compromised in a rat model of depression. J Psychiatr Res 2013; 47: 579–585. 23421961 10.1016/j.jpsychires.2013.01.006

[bib56] Wayman GA, Lee YS, Tokumitsu H, Silva AJ, Soderling TR. Calmodulin-kinases: modulators of neuronal development and plasticity. Neuron 2008; 59: 914–931. 18817731 10.1016/j.neuron.2008.08.021 PMC2664743

[bib57] Novak G, Seeman P, Tallerico T. Increased expression of calcium/calmodulin-dependent protein kinase IIbeta in frontal cortex in schizophrenia and depression. Synapse 2006; 59: 61–68. 16247765 10.1002/syn.20211

[bib58] Dwivedi Y, Rizavi HS, Conley RR, Roberts RC, Tamminga CA, Pandey GN. Altered gene expression of brain-derived neurotrophic factor and receptor tyrosine kinase B in postmortem brain of suicide subjects. Arch Gen Psychiatry 2003; 60: 804–815. 12912764 10.1001/archpsyc.60.8.804

[bib59] Dwivedi Y, Rizavi HS, Pandey GN. Antidepressants reverse corticosterone-mediated decrease in brain-derived neurotrophic factor expression: differential regulation of specific exons by antidepressants and corticosterone. Neuroscience 2006; 139: 1017–1029. 16500030 10.1016/j.neuroscience.2005.12.058 PMC1513636

[bib60] Dwivedi Y, Rao JS, Rizavi HS, Kotowski J, Conley RR, Roberts RC et al. Abnormal expression and functional characteristics of cyclic adenosine monophosphate response element binding protein in postmortem brain of suicide subjects. Arch Gen Psychiatry 2003; 60: 273–282. 12622660 10.1001/archpsyc.60.3.273

[bib61] Murase S, Schuman EM. The role of cell adhesion molecules in synaptic plasticity and memory. Curr Opin Cell Biol 1999; 11: 549–553. 10508654 10.1016/s0955-0674(99)00019-8

[bib62] Skaper SD. The neurotrophin family of neurotrophic factors: an overview. Methods Mol Biol 2012; 846: 1–12. 22367796 10.1007/978-1-61779-536-7_1

[bib63] Dwivedi Y, Rizavi HS, Zhang H, Roberts RC, Conley RR, Pandey GN. Modulation in activation and expression of phosphatase and tensin homolog on chromosome ten, Akt1, and 3-phosphoinositide-dependent kinase 1: further evidence demonstrating altered phosphoinositide 3-kinase signaling in postmortem brain of suicide subjects. Biol Psychiatry 2010; 67: 1017–10125. 20163786 10.1016/j.biopsych.2009.12.031 PMC2868089

[bib64] Wang ZZ, Zhang Y, Liu YQ, Zhao N, Zhang YZ, Yuan L et al. RNA interference-mediated phosphodiesterase 4D splice variants knock-down in the prefrontal cortex produces antidepressant-like and cognition-enhancing effects. Br J Pharmacol 2013; 168: 1001–1014. 23003922 10.1111/j.1476-5381.2012.02225.x PMC3631387

[bib65] Leonard BE. The HPA and immune axes in stress: the involvement of the serotonergic system. Eur Psychiatry 2005; 20: S302–S306. 16459240 10.1016/s0924-9338(05)80180-4

[bib66] Bouvette-Turcot AA, Fleming AS, Wazana A, Sokolowski MB, Gaudreau H, Gonzalez A et al. Maternal childhood adversity and child temperament: an association moderated by child 5-HTTLPR genotype. Genes Brain Behav 2015; 14: 229–237. 25688466 10.1111/gbb.12205

[bib67] Poulter MO, Du L, Weaver ICG, Palkovits M, Faludi G, Merali Z et al. GABAA receptor promoter hypermethylation in suicide brain: implications for the involvement of epigenetic processes. Biol Psychiatry 2008; 64: 645–652. 18639864 10.1016/j.biopsych.2008.05.028

[bib68] Zimmermann CA, Hoffmann A, Raabe F, Spengler D. Role of mecp2 in experience-dependent epigenetic programming. Genes (Basel) 2015; 6: 60–86. 25756305 10.3390/genes6010060 PMC4377834

[bib69] Lercher MJ, Urrutia AO, Hurst LD. Clustering of housekeeping genes provides a unified model of gene order in the human genome. Nat Genet 2002; 3: 180–183. 10.1038/ng887 11992122

[bib70] Landgraf P, Rusu M, Sheridan R, Sewer A, Iovino N, Aravin A et al. A mammalian microRNA expression atlas based on small RNA library sequencing. Cell 2007; 129: 1401–1414. 17604727 10.1016/j.cell.2007.04.040 PMC2681231

[bib71] Maiorano NA, Mallamaci A. The pro-differentiating role of miR-124: indicating the road to become a neuron. RNA Biol 2010; 7: 528–533. 20523124 10.4161/rna.7.5.12262

[bib72] Rajasethupathy P, Fiumara F, Sheridan R, Betel D, Puthanveettil SV, Russo JJ et al. Characterization of small RNAs in Aplysia reveals a role for miR-124 in constraining synaptic plasticity through CREB. Neuron 2009; 63: 803–817. 19778509 10.1016/j.neuron.2009.05.029 PMC2875683

[bib73] Vreugdenhil E, Verissimo CS, Mariman R, Kamphorst JT, Barbosa JS, Zweers T et al. MicroRNA 18 and 124a down-regulate the glucocorticoid receptor: implications for glucocorticoid responsiveness in the brain. Endocrinology 2009; 150: 2220–2228. 19131573 10.1210/en.2008-1335

[bib74] Unemura K, Kume T, Kondo M, Maeda Y, Izumi Y, Akaike A. Glucocorticoids decrease astrocyte numbers by reducing glucocorticoid receptor expression *in vitro* and *in vivo*. J Pharmacol Sci 2012; 119: 30–39. 22641130 10.1254/jphs.12047fp

[bib75] Cao MQ, Chen DH, Zhang CH, Wu ZZ. [Screening of specific microRNA in hippocampus of depression model rats and intervention effect of Chaihu Shugan San]. Zhongguo Zhong Yao Za Zhi 2013; 38: 1585–1589. 23947143

[bib76] Szczepankiewicz A, Leszczyńska-Rodziewicz A, Pawlak J, Narozna B, Rajewska-Rager A, Wilkosc M et al. FKBP5 polymorphism is associated with major depression but not with bipolar disorder. J Affect Disord 2014; 164: 33–37. 24856550 10.1016/j.jad.2014.04.002

[bib77] Menke A, Klengel T, Rubel J, Brückl T, Pfister H, Lucae S et al. Genetic variation in FKBP5 associated with the extent of stress hormone dysregulation in major depression. Genes Brain Behav 2013; 12: 289–296. 23406438 10.1111/gbb.12026

[bib78] Hoeijmakers L, Harbich D, Schmid B, Lucassen PJ, Wagner KV, Schmidt MV et al. Depletion of FKBP51 in female mice shapes HPA axis activity. PLoS One 2014; 9: e95796. 24759731 10.1371/journal.pone.0095796 PMC3997427

[bib79] Rinaldi A, Vincenti S, De Vito F, Bozzoni I, Oliverio A, Presutti C et al. Stress induces region specific alterations in microRNAs expression in mice. Behav Brain Res 2010; 208: 265–269. 19913057 10.1016/j.bbr.2009.11.012

