# Supplementary material for: Chronic corticosterone-mediated dysregulation of microRNA network in prefrontal cortex of rats: relevance to depression pathophysiology
Source: Transl Psychiatry. 2015 Nov 17;5(11):e682–. doi: 10.1038/tp.2015.175 (PMC5068767; doi:10.1038/tp.2015.175)
Supplement: Supplementary Table 1 [file tp2015175x1.doc]

| **Supplemental Table 1. Number of animals used for each assay** | | |
| --- | --- | --- |
| **Behavioral procedures/miRNA studies** | **CORT treatment** | **Number of animals** |
| Sucrose preference test | 50 mg/kg for 21 days and continued for additional 3 days for sucrose preference testing. | 8 Vehicle treated  8 CORT treated |
| Forced swim test | 50 mg/kg for 21 days and continued for additional 2 days for swim testing. | 8 Vehicle treated  8 CORT treated |
| Open field | 50 mg/kg for 21 days. | 8 Vehicle treated  8 CORT treated |
| miRNA expression | 50 mg/kg for 21 days. | 8 Vehicle treated (pooled to 4 samples)  8 CORT treated (pooled to 4 samples) |
| Serum CORT level (in the same samples in which miRNA expression was determined) | 50 mg/kg for 21 days. | 8 Vehicle treated  8 CORT treated |
