# Supplementary material for: Chronic corticosterone-mediated dysregulation of microRNA network in prefrontal cortex of rats: relevance to depression pathophysiology
Source: Transl Psychiatry. 2015 Nov 17;5(11):e682–. doi: 10.1038/tp.2015.175 (PMC5068767; doi:10.1038/tp.2015.175)
Supplement: Supplementary Table 4 [file tp2015175x4.doc]

|  |  | | | | | |
| --- | --- | --- | --- | --- | --- | --- |
| **Supplemental Table 4. Ct and ΔΔCT values of a select target genes examined as qPCR based target gene expression assay at transcript level** | | | | | | |
| **Genes** | | **Groups** | **Mean Ct** | **U6 Mean Ct** | **ΔCt** | **ΔΔCt** |
| rno-miR-124 | | VEH | 23.31 | 17.60 | 5.71 |  |
|  |  | CORT | 22.25 | 17.20 | 5.05 | -0.66 |
| rno-miR-218 | | VEH | 21.52 | 17.60 | 3.92 |  |
|  |  | CORT | 20.72 | 17.20 | 3.52 | -0.40 |
| rno-miR-29a | | VEH | 19.64 | 17.60 | 2.04 |  |
|  |  | CORT | 18.61 | 17.20 | 1.41 | -0.63 |
| rno-miR-146a | | VEH | 26.70 | 17.60 | 9.1 |  |
|  |  | CORT | 27.15 | 17.20 | 9.95 | -0.85 |
| rno-miR-200c | | VEH | 28.88 | 17.60 | 11.28 |  |
|  |  | CORT | 31.17 | 17.20 | 13.97 | 2.69 |
| rno-miR-155 | | VEH | 28.13 | 17.60 | 10.53 |  |
|  |  | CORT | 28.91 | 17.20 | 11.71 | 1.18 |
