# Supplementary material for: Chronic corticosterone-mediated dysregulation of microRNA network in prefrontal cortex of rats: relevance to depression pathophysiology
Source: Transl Psychiatry. 2015 Nov 17;5(11):e682–. doi: 10.1038/tp.2015.175 (PMC5068767; doi:10.1038/tp.2015.175)
Supplement: Supplementary Table 5 [file tp2015175x5.doc]

| **Supplemental Table 5. Ct and ΔΔCT values of a select target genes examined as qPCR based target gene expression assay at transcript level** | | | | |
| --- | --- | --- | --- | --- |
| **Genes** | **Groups** | **Mean Ct** | **ΔCt** | **ΔΔCt** |
| CREB1 | VEH | 23.05 | 2.59 |  |
|  | CORT | 24.52 | 4.30 | 1.72 |
| BDNF | VEH | 24.30 | 3.84 |  |
|  | CORT | 25.43 | 5.23 | 1.39 |
| CaMKIIα | VEH | 29.33 | 8.87 |  |
|  | CORT | 30.55 | 10.34 | 1.47 |
| PTEN | VEH | 28.18 | 7.72 |  |
|  | CORT | 27.93 | 7.73 | 0.00166 |
| AKT3 | VEH | 23.55 | 3.09 |  |
|  | CORT | 24.85 | 4.64 | 1.55 |
| NR3C1 | VEH | 26.23 | 5.77 |  |
|  | CORT | 27.20 | 6.99 | 1.22 |
| VEGFA | VEH | 27.02 | 6.56 |  |
|  | CORT | 27.23 | 7.03 | 0.47 |

|  |
| --- |
|  |
|  |
|  |
|  |
|  |
|  |
|  |
|  |
|  |
|  |
|  |
|  |
|  |
