# Supplementary material for: Chronic corticosterone-mediated dysregulation of microRNA network in prefrontal cortex of rats: relevance to depression pathophysiology
Source: Transl Psychiatry. 2015 Nov 17;5(11):e682–. doi: 10.1038/tp.2015.175 (PMC5068767; doi:10.1038/tp.2015.175)
Supplement: Supplementary Table 6 [file tp2015175x6.doc]

| **Supplemental Table 6. CORT-mediated altered miRNAs and predicated functionally relevant target genes** | |
| --- | --- |
| **MicroRNA** | **Functionally Relevant Specific Target Genes** |
| let-7i | AKAP5, AKAP6, AKAP8, ELK4, ESR2, GRIK2, GRIN3A, IL10, IL6, MAP3K2, MEF2C, NGF, PLCB2, Ras, SLC1A4, SLC38A1, SLC6A1, TLR4, TNFAIP3 |
| miR-101a | AKAP11, AKT3, CREB1, DNMT3A, FOXO1, GABRA1, GSK3B, IL1R1, MAOA, MAPK1, MTOR, NAIP, PDE10A, PDE4A, PDE4D, PIK3C2A, PIK3C2B, RAC1, RAP1B, RAP2B, SLC1A1 |
| miR-124 | AKAP13, AKAP6, AKT3, BDNF, CREB1, DNMT3B, EIF4EBP2, ETS1, GRIA2, GRIA3, GRIA4, GSK3B, HDAC4, HDAC5, HDAC9, IL10RA, IL6R, IL9R, IRAK3, MAPK1, NR3C1, NR3C2, PDE2A, PDE4A, PDE4B, PDE4D, PDE5A, PDE7A, PIK3C2A, PLCB1, SLC1A4, EPHA10, ACTC1 |
| miR-137 | AKAP2, BMP7, BRAF, CACNA1C, CAMK2A, FKBP4, GABRA1, GRIA1, GRIA4, GSK3B, PDE4A, PDE4D, PLCB1, SLC6A1, SP1, TNF, TNFAIP6 |
| miR-146a | BRK1, RAC1, TLR1, TLR10, TLR4, TLR9, ACTC1 |
| miR-153 | BAD, BCL2, EPHA10, ACTC1 |
| miR-155 | PRKAR1A, PRKAR1B |
| miR-181c | AKT3, CEBPA, CREB1, EIF4A2, GABRA1, GRM7, HSP90B1, IL1A, IL2, MAPK1, MARCKS, PDE10A, PDE5A, PIK3C2A, PTEN, TGFBR1, TNFAIP6 |
| miR-19b | AKAP1, AKAP7, CACNA1C, GRIN2A, GSK3B, MECP2, MEF2C, PDE4A,, SLC6A4, TRPC3, WNT1, WNT10A, WNT3, WNT7B, ACTC1, TNFAIP3 |
| miR-200c | DNMT3A, DNMT3B, HSPA13, HSPA9, LIMK1, PIP4K2A, PIP4K2B, SLIT1, ACTC1 |
| miR-203 | CREB1, CREM, EIF4E, FKBP5, GABRA1, HTR2A, HTR2C, RGS7, TLR4, VEGFA, ABL1 |
| miR-218 | CREB1, EGFR, GRIA2, GRIA4, GRIK2, GRIK3, GSK3B, SEMA4A, SEMA5A, SEMA6A, SEMA6B, SLC38A1, SLC6A3, TRPC3, TRPC6 |
| miR-29a | AKT3, DNMT3A, DNMT3B, EIF2S2, ELF2, FKBP4, FOXO3, GRM4, GSK3B, NR3C1, PIK3R1, PIK3R3, PTEN, SLC1A3, VEGFA, EPHA10, ACTC1, TNFAIP3 |
| miR-30e | BCL2, BDNF, CAMK4, DNMT3A, HDAC5, IL1A, MARCKS, MECP2, NR3C1, NR3C2, PDE4D, PDE5A, PDE7A, RAP1B, RAP2B, RGS2, SLC38A1, TSC1, ABL1, ACTC1 |
| miR-324-5p | CAMK2B, EIF4EBP2, ESR1, GRIK1, PLD1, RAP1A, SLC6A4 |
| miR-351 | BAK1, EIF4EBP1, ELK1, LIMK1, NFATC1, NTF4, RAF1, SLC18A3, VEGFA, ABL1, EPHA10, PLCB3, ACTC1 |
| miR-365 | AKT3, BAX, BCL2, BDNF, CACNA1C, ETS1, GRIN3A, IL1A, IL6, MAPK1, MECP2, NR3C1, NR3C2, PDE4D, PLCB4, SLC1A3, TLR4, EPHA10, ACTC1 |
| miR-409-5p | EIF4EBP2, MARCKS, NAIP, CREB1, ESR2, NR3C1, PDE7A, RGS7, TNFAIP3 |
| miR-721 | AKAP1, AKAP11, AKAP7, CREB1, FKBP5, GABRA1, GABRB2, GRIK2, MAPK1, PDE4D, PLCB1, PTEN, TGFBR1, EPHA10 |
