# Supplementary material for: Chronic corticosterone-mediated dysregulation of microRNA network in prefrontal cortex of rats: relevance to depression pathophysiology
Source: Transl Psychiatry. 2015 Nov 17;5(11):e682–. doi: 10.1038/tp.2015.175 (PMC5068767; doi:10.1038/tp.2015.175)
Supplement: Supplementary Table 7 [file tp2015175x7.doc]

| **Supplemental Table 7. Genes affected by multiple CORT-mediated altered miRNAs** | | |
| --- | --- | --- |
| **Genes** | **miRNAs** | **Prediction confidence** |
| AKT3 | miR-101a | High |
| AKT3 | miR-124 | Moderate |
| AKT3 | miR-181c | Moderate |
| AKT3 | miR-29a | High |
| AKT3 | miR-365 | High |
| BCL2 | miR-153 | Experimentally observed |
| BCL2 | miR-30e | High |
| BCL2 | miR-365 | High |
| BDNF | miR-124 | Experimentally observed |
| BDNF | miR-30e | High |
| BDNF | miR-365 | High |
| CREB1 | miR-101a | High |
| CREB1 | miR-124 | High |
| CREB1 | miR-721 | High |
| CREB1 | miR-181c | High |
| CREB1 | miR-203 | High |
| CREB1 | miR-218 | High |
| CREB1 | miR-582-5p | Moderate |
| CREB1 | miR-351 | High |
| CREB1 | miR-155 | High |
| CREB1 | miR-200c | High |
| DNMT3A | miR-200c | High |
| DNMT3A | miR-101a | High |
| DNMT3A | miR-29a | Experimentally observed |
| DNMT3A | miR-30e | High |
| ETS1 | miR-351 | High |
| ETS1 | miR-155 | Experimentally observed |
| ETS1 | miR-200c | High |
| GABRA1 | miR-101a | Moderate |
| GABRA1 | miR-721 | Moderate |
| GABRA1 | miR-137 | High |
| GABRA1 | miR-181c | High |
| GABRA1 | miR-155 | High |
| GABRA1 | miR-203 | High |
| GRIA4 | miR-124 | High |
| GRIA4 | miR-137 | High |
| GRIA4 | miR-218 | High |
| GSK3B | miR-155 | High |
| GSK3B | miR-101a | High |
| GSK3B | miR-124 | High |
| GSK3B | miR-137 | High |
| GSK3B | miR-19b | High |
| GSK3B | miR-218 | High |
| GSK3B | miR-29a | High |
| MAPK1 | miR-101a | High |
| MAPK1 | miR-124 | High |
| MAPK1 | miR-721 | High |
| MAPK1 | miR-181c | High |
| MAPK1 | miR-365 | High |
| NR3C1 | miR-29a | Moderate to high |
| NR3C1 | miR-30e | High |
| NR3C1 | miR-365 | High |
| NR3C1 | miR-582-5p | Moderate |
| NR3C2 | miR-124 | High |
| NR3C2 | miR-30e | High |
| NR3C2 | miR-365 | High |
| PDE4A | miR-101a | High |
| PDE4A | miR-124 | High |
| PDE4A | miR-137 | High |
| PDE4A | miR-19b | High |
| PDE4D | miR-101a | High |
| PDE4D | miR-124 | High |
| PDE4D | miR-721 | High |
| PDE4D | miR-137 | High |
| PDE4D | miR-30e | High |
| PDE4D | miR-365 | High |
| Tragetscan, miRecords, Ingenuity Expert Finding, and Tarbase were used for the prediction analysis. | | |
