# Supplementary figures and images for: Chronic corticosterone-mediated dysregulation of microRNA network in prefrontal cortex of rats: relevance to depression pathophysiology
Source: Transl Psychiatry. 2015 Nov 17;5(11):e682–. doi: 10.1038/tp.2015.175 (PMC5068767; doi:10.1038/tp.2015.175)

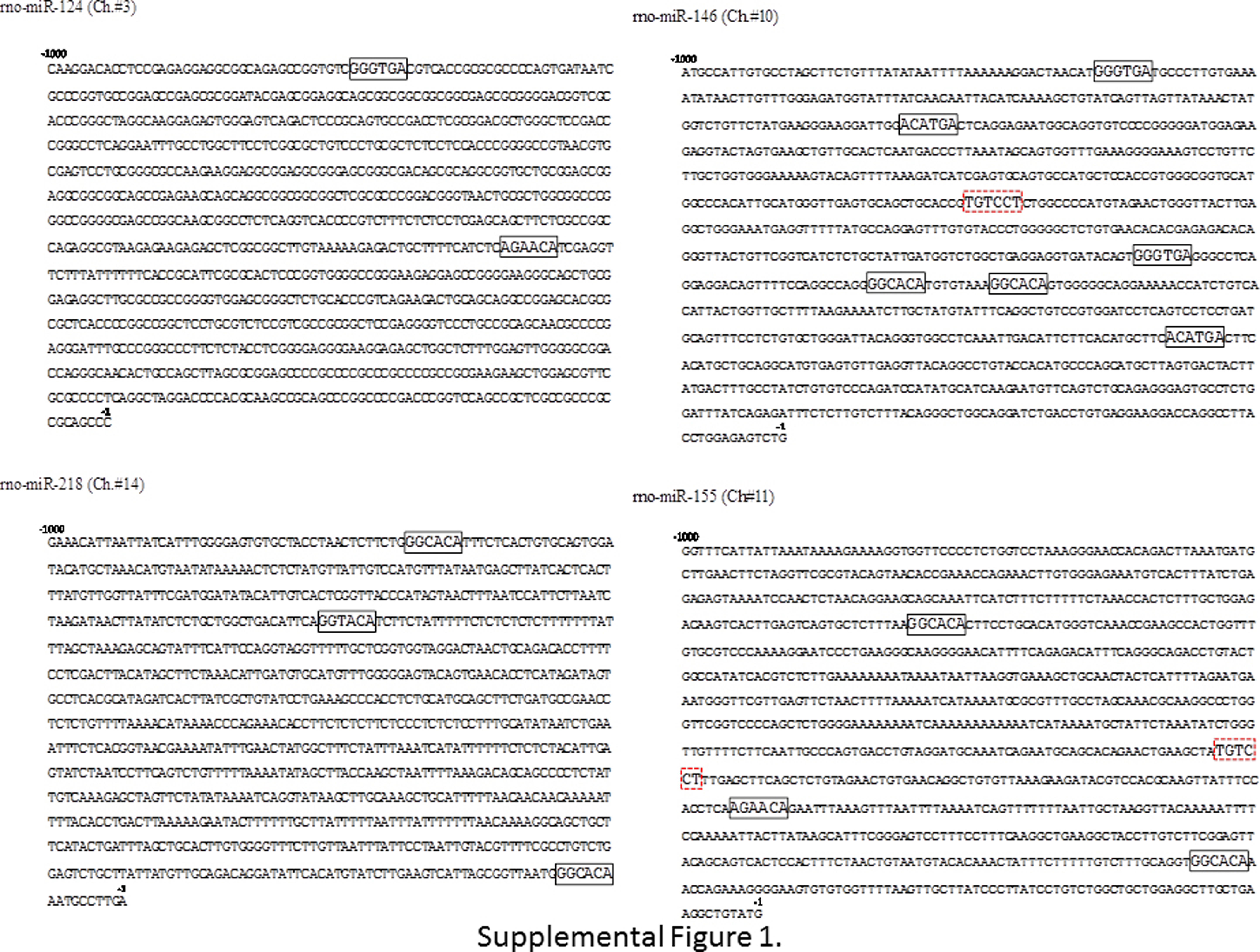

Supplement: Supplementary Figure 1 [file tp2015175x8.tif]

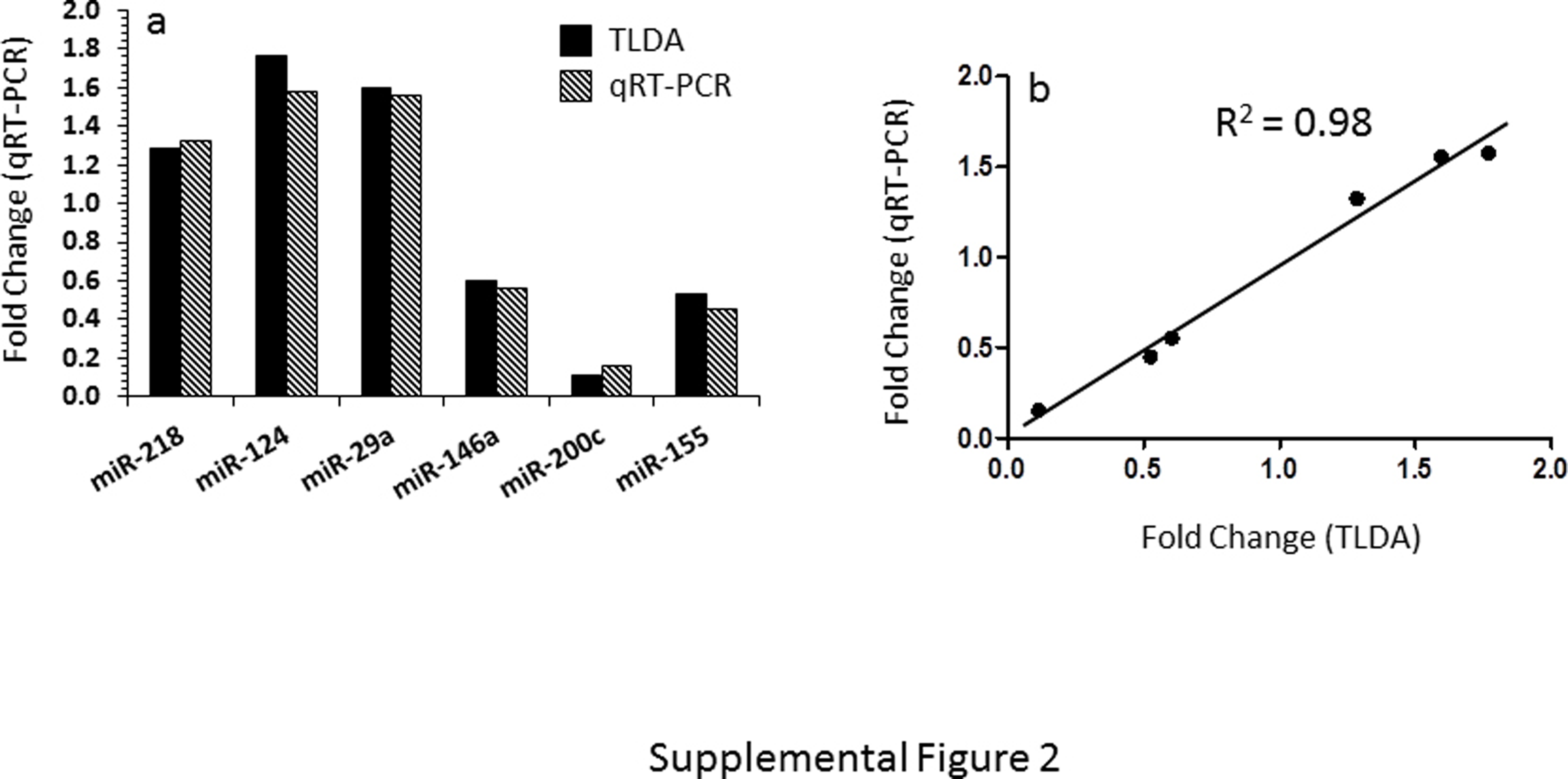

Supplement: Supplementary Figure 2 [file tp2015175x9.tif]

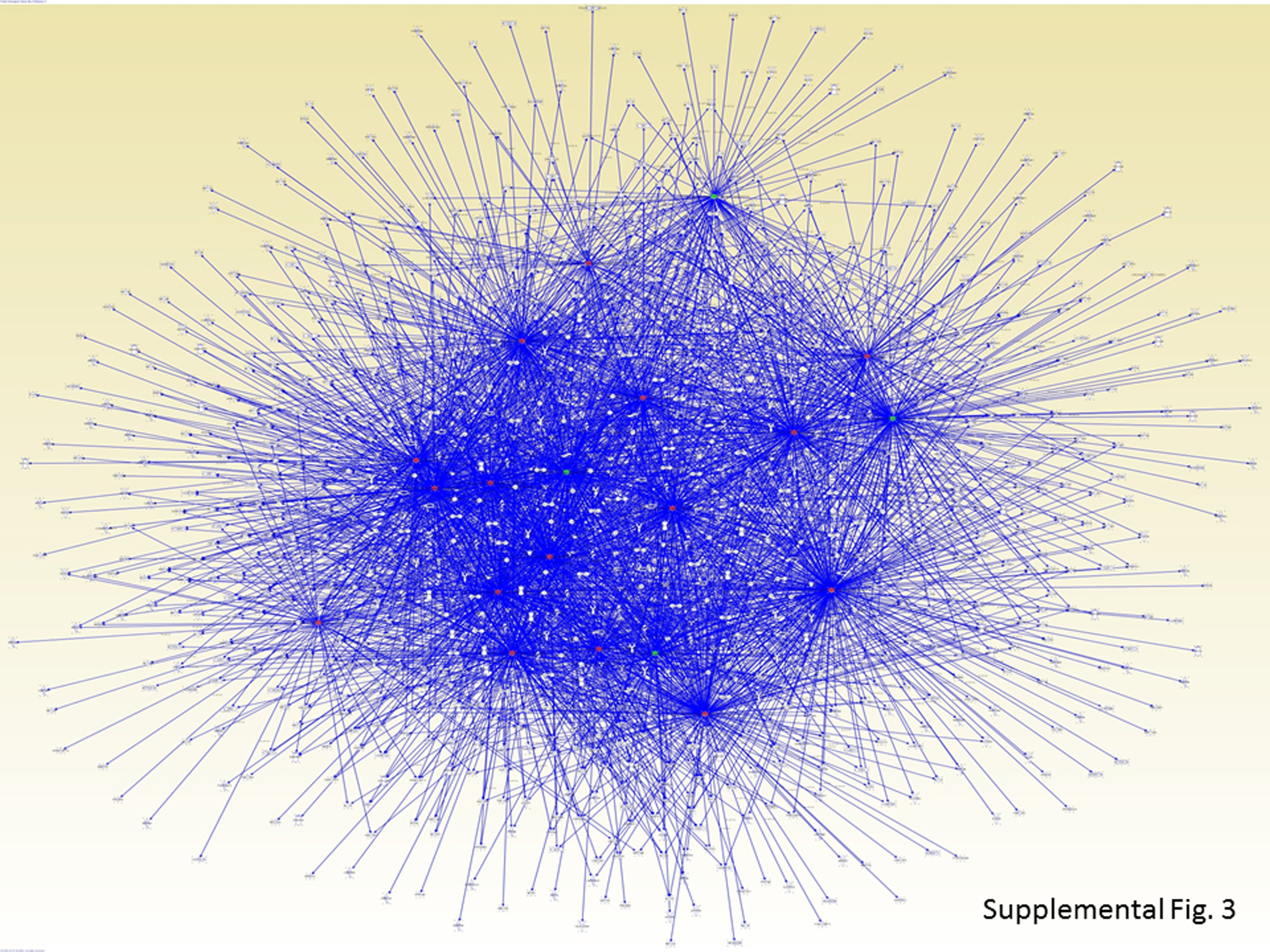

Supplement: Supplementary Figure 3 [file tp2015175x10.tif]

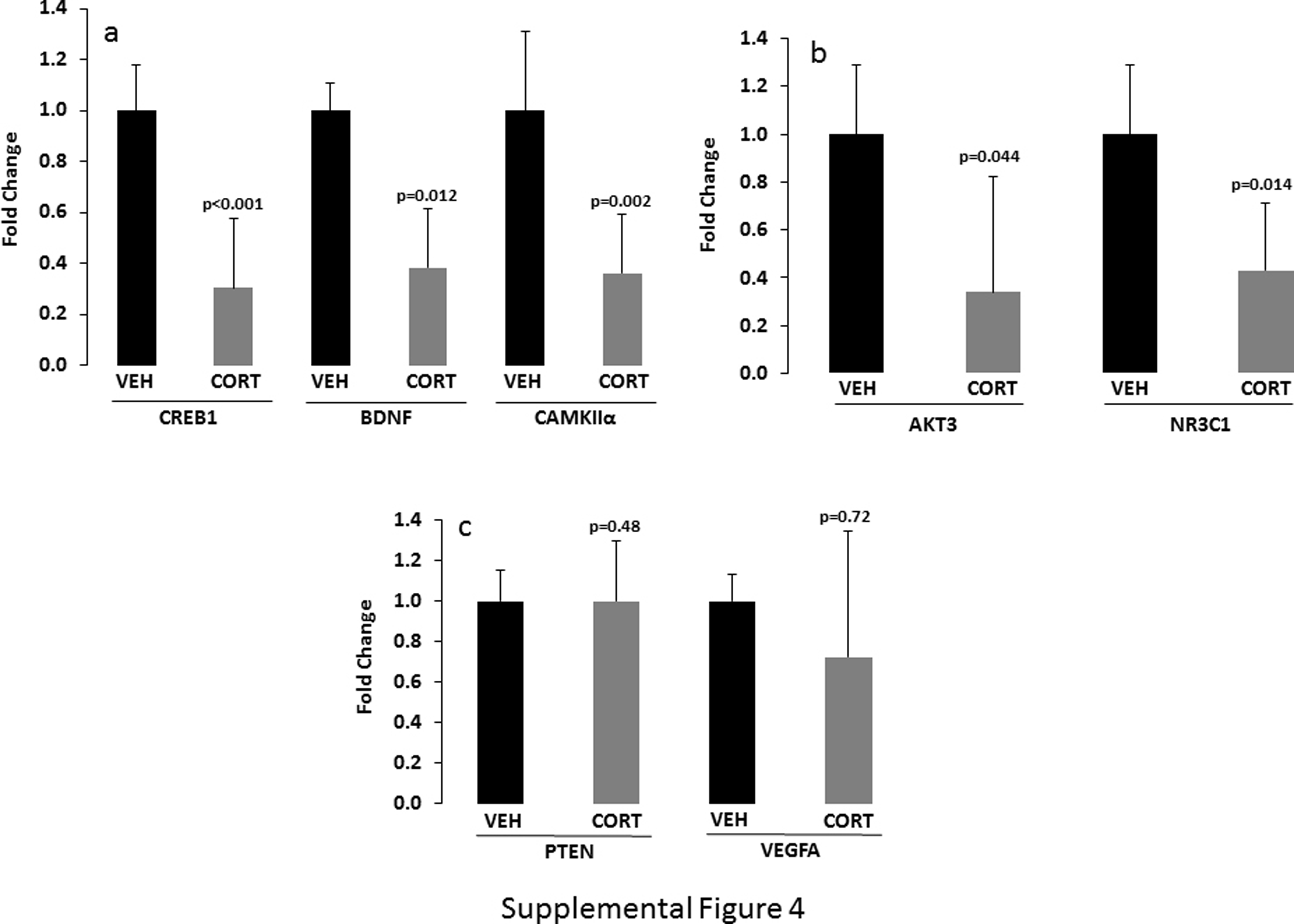

Supplement: Supplementary Figure 4 [file tp2015175x11.tif]
