# Supplementary material for: Chronic corticosterone-mediated dysregulation of microRNA network in prefrontal cortex of rats: relevance to depression pathophysiology
Source: Transl Psychiatry. 2015 Nov 17;5(11):e682–. doi: 10.1038/tp.2015.175 (PMC5068767; doi:10.1038/tp.2015.175)
Supplement: Supplementary Information [file tp2015175x12.doc]

**Legends for Supplemental Figures and Tables**

**Supplemental Figure 1: Mapping of putative glucocorticoid receptor element (GRE) half sites on four significantly altered rat miRNA promoter elements.** Mapping of both positive (half site demarcated with solid black box) and negative (half site demarcated with broken red box) GREs on the proximal promoter region (up to 1kb upstream) of significantly altered four miRNAs (miR-124, Mir-218, miR-146, miR-155) under chronic CORT treatment unfolds the abundance and relative position to the transcription start site (TSS). -1 base denotes the penultimate nucleotide to TSS.

**Supplemental Figure 2: Validation of a few selected CORT-mediated altered miRNAs.** (**a**) Relative abundance of selected miRNAs reanalyzed by q-PCR and their comparison with miRNA measurements done by TLDA assay is shown. Sequences for oligo primers for these miRNAs are provided in **the Methods section.** (**b**) Correlation between miRNAs measured by q-PCR and TLDA. Data are the mean ± SD. qPCR of miRNAs was analyzed in 8 rats per group.

**Supplemental Figure 3: Gene network analysis based on CORT-mediated altered miRNAs.** Significantly altered miRNAs were analyzed for their mRNA targets using Ingenuity Pathway Analysis Software (IPA). MiRNA target module in IPA was used to filter the list of significantly altered up or down regulated miRNAs to retrieve a population of target mRNA. mRNA target gene list was prepared using Tar Base, Ingenuity Expert Finding, miRecords, as well as number of conserved targeting sites indicated in the Methods section. Total context score prediction value with a high to moderate degree of 3’UTR binding specificity with the miRNA seed sequences were also considered. Green color denotes the upregulated miRNAs and red color denotes the downregulated ones.

**Supplemental Figure 4: Validation of a few select target genes for upregulated miRNAs.** mRNA levels of CREB1, BDNF, CaMKIIα, PTEN, AKT3, NR3C1, and VEGFA were analyzed in prefrontal cortex of CORT- and vehicle-treated rats by qPCR using primers mentioned in the Methods section. Figure 4a represents the relative transcript quantification for gene CREB1, BDNF and CaMKIIα, Figure 4b is the representation of relative gene expression levels for AKT3 and NR3C1. The level of significance has been indicated on top of each column representing the CORT treatment group. In Figure 4c the relative gene expression for PTEN and VEGFA have been mentioned, no significance value has been indicated for these two genes as they were found not be significantly altered. Values were normalized using GAPDH. Data are the mean ± SEM from 6 rats per group.

**Supplemental Table 1:** Number of animals used in each assay.

**Supplemental Table 2:** List of all miRNAs analyzed by TLDA plates A and B. The miRNA which showed Ct ≤ 35 are marked as bold face.

**Supplemental Table 3:** Mean expression level of significantly altered miRNAs from TLDA assay (represented as Ct values). The ΔCT and ΔΔCT values for these miRNAs are also mentioned in this table.

**Supplemental Table 4:** Ct and ΔΔCT values of six miRNAs examined as qPCR based validation assay.

**Supplemental Table 5:** Ct and ΔΔCT values of a select target genes examined as qPCR based target gene expression assay at transcript level.

**Supplemental Table 6:** Genes affected by CORT-mediated altered miRNAs.

**Supplemental Table 7:** List of predicted target genes for CORT-mediated altered miRNAs.
